# *Agaricus subrufescens* fermented rye affects the development of intestinal microbiota, local intestinal and innate immunity in suckling-to-nursery pigs

**DOI:** 10.1186/s42523-023-00244-w

**Published:** 2023-04-11

**Authors:** Caifang Wen, Mirelle Geervliet, Hugo de Vries, Lluís Fabà, Petra J. Roubos-van den Hil, Kerstin Skovgaard, Huub F. J. Savelkoul, Henk A. Schols, Jerry M. Wells, Edwin Tijhaar, Hauke Smidt

**Affiliations:** 1 Laboratory of Microbiology, Wageningen University & Research, Wageningen, The Netherlands; 2 Laboratory of Food Chemistry, Wageningen University & Research, Wageningen, The Netherlands; 3 Cell Biology and Immunology Group, Wageningen University & Research, Wageningen, The Netherlands; 4 Host-Microbe Interactomics Group, Wageningen University & Research, Wageningen, The Netherlands; 5 Research and Development, Trouw Nutrition, Amersfoort, The Netherlands; 6 Department of Biotechnology and Biomedicine, Technical University of Denmark, Lyngby, Denmark; 7 Present Address: DSM Food and Beverages – Fresh Dairy, Wageningen, The Netherlands

**Keywords:** Pigs, *Agaricus subrufescens*, Gut microbiota, Immunity, Early life, Fermentation

## Abstract

**Background:**

*Agaricus subrufescens* is considered as one of the most important culinary-medicinal mushrooms around the world. It has been widely suggested to be used for the development of functional food ingredients to promote human health ascribed to the various properties (e.g., anti-inflammatory, antioxidant, and immunomodulatory activities). In this context, the interest in *A. subrufescens* based feed ingredients as alternatives for antibiotics has also been fuelled during an era of reduced/banned antibiotics use. This study aimed to investigate the effects of a fermented feed additive -rye overgrown with mycelium (ROM) of *A. subrufescens—*on pig intestinal microbiota, mucosal gene expression and local and systemic immunity during early life. Piglets received ROM or a tap water placebo (Ctrl) perorally every other day from day 2 after birth until 2 weeks post-weaning. Eight animals per treatment were euthanized and dissected on days 27, 44 and 70.

**Results:**

The results showed ROM piglets had a lower inter-individual variation of faecal microbiota composition before weaning and a lower relative abundance of proteobacterial genera in jejunum (*Undibacterium* and *Solobacterium)* and caecum (*Intestinibacter* and *Succinivibrionaceae*_UCG_001) on day 70, as compared to Ctrl piglets. ROM supplementation also influenced gut mucosal gene expression in both ileum and caecum on day 44. In ileum, ROM pigs showed increased expression of TJP1/ZO1 but decreased expression of CLDN3, CLDN5 and MUC2 than Ctrl pigs. Genes involved in TLR signalling (e.g., TICAM2, IRAK4 and LY96) were more expressed but MYD88 and TOLLIP were less expressed in ROM pigs than Ctrl animals. NOS2 and HIF1A involved in redox signalling were either decreased or increased in ROM pigs, respectively. In caecum, differentially expressed genes between two groups were mainly shown as increased expression (e.g., MUC2, PDGFRB, TOLLIP, TNFAIP3 and MYD88) in ROM pigs. Moreover, ROM animals showed higher NK cell activation in blood and enhanced IL-10 production in ex vivo stimulated MLN cells before weaning.

**Conclusions:**

Collectively, these results suggest that ROM supplementation in early life modulates gut microbiota and (local) immune system development. Consequently, ROM supplementation may contribute to improving health of pigs during the weaning transition period and reducing antibiotics use.

**Supplementary Information:**

The online version contains supplementary material available at 10.1186/s42523-023-00244-w.

## Introduction

Pigs are exposed to various stressors during their early life in modern commercial farming, with weaning as one of the most stressful events in a pig’s life. The weaning associated psychological, environmental and nutritional stressors have the potential to disrupt the pig’s gastrointestinal (GI) health, increase disease susceptibility, thereby compromising animal health, welfare and growth performance [1]. Recently, there is an increasing interest in fermented feed additives as dietary alternatives for antibiotics to ameliorate the weaning associated GI dysfunction in an era of reduced/banned antibiotics use [2, 3]. Those studies on fermented feed additives suggest positive effects on animal gut health, nutrient uptake ability, and immune function.

One of these additives is rye overgrown with mycelium (ROM) of *Agaricus subrufescens* (*A. subrufescens*) through the process of solid-state fermentation. During the fermentation procedure, *A. subrufescens* produces enzymes (e.g., xylanases, glucanases and a-amylases) that assist in the degradation and utilization of substrate (rye) nutrients (e.g., arabinoxylan) into indigestible but bioactive oligosaccharides [4, 5]. Meanwhile, a range of fungal cell components and metabolites are produced that have been shown to be potentially beneficial and bioactive including polysaccharides (e.g., 1,3-1,6-β-glucans) and phenolic compounds (e.g., gallic acid and syringic acid) [6]. Human clinical studies on the immunomodulatory activities of mainly *A. subrufescens* mycelium based extract (Andosan™) were reviewed by Hetland et al. [7]. Moreover, *A. subrufescens* material has even been recently proposed as supplement for prophylactic or therapeutic treatment in COVID‐19 infection to aid against pneumococcal superinfection*,* immune overreaction, and damaging inflammation [8, 9]. Nonetheless, only limited research has been performed to evaluate the impact of *A. subrufescens*-based product on piglet health. Fabà et al. reported that feeding ROM combined with organic acids to nursery pigs reduced *S. Typh* shedding over a 21-day period post-challenge [10]. De Groot et al. observed that inclusion of a blend of ROM combined with mannan-rich hydrolysed copra meal for nursery pigs exerted immunomodulatory effects both in the GI tract and at systemic level [11]. Recently, the neonatal period (spanning birth to weaning) has been described as a critical “window of opportunity” that allows for an important cross-talk between gut microbiota and immune system [12, 13]. If the host–microbe cross-talk is perturbed early in life, an increased susceptibility to disease may develop later in life [13, 14]. It is hypothesized that modulating the gut microbiota and enhancing host immune system development early in life may enable pigs to be more resilient towards challenges they will encounter during their lifetime.

Therefore, the aim of the present study was to assess potential impacts of ROM on various aspects of GI tract health and immune system development starting from the suckling period in young piglets. To this end, a pig in vivo trial was conducted, starting from birth onwards to 70 days of age. We hypothesized that early life supplementation of ROM induces changes in the development of the intestinal microbiota as well as the immune system in early life, which may improve the host immune competence and thus enable piglets to better cope with the weaning transition.

## Methods

The animal experiment was conducted in accordance with Dutch law, and the Dutch Central Authority for Scientific Procedures on Animals (CCD) approved the experiment under license number AVD1040020173948. Furthermore, animal management and experimental procedures were approved by the Animal Care and Use Committee of Wageningen University & Research (Wageningen, The Netherlands).

### Experimental design and procedures

To investigate the effect of ROM on early life GI development and immune function in pigs, 33 sows (Hypor Libra, Boxmeer, The Netherlands) and their litters (Maxter × Hypor Libra sow) were used at the Swine Research Centre (Trouw Nutrition, Sint Anthonis, The Netherlands). All sows got inseminated from a single boar with the aim of reducing genetic variation in the litters. After parturition, the piglets immediately received an ear tag and an intramuscular iron injection, and their birth weight and sex were recorded. Piglet tails were docked as corrective procedure to prevent tail biting. To lower the risk of infection pressure in the stable, Calcium carbonate powder (Power-Cal®, Power-Cal, Sint-Oedenrode, The Netherlands) was spread to all pen floors. One day after parturition, 192 female piglets were selected and cross-fostered to minimize possible confounding (e.g., birth weight, sow parity and litter size). Of these 192 female piglets, a total of 96 piglets were randomly assigned to either treatment (ROM) or control (Ctrl) groups, while groups were matched in terms of sow parity (Ctrl: average parity 3.88, ranging from 1 to 7; ROM: average parity 3.38, ranging from 1 to 6) and birth body weight (Ctrl:1.47 ± 0.3 kg; ROM: 1.46 ± 0.29 kg). Six female piglets per pen with 16 pens (and sows) and 48 piglets per treatment were distributed over four farrowing rooms. Pen was the experimental unit and contained either ROM or Ctrl piglets. Males and female piglets that were not included in the experiment were also equally divided over pens and housed together with experimental piglets, leading to an average number of 12 piglets per pen at the start of the experiment.

**Fig. 1:**
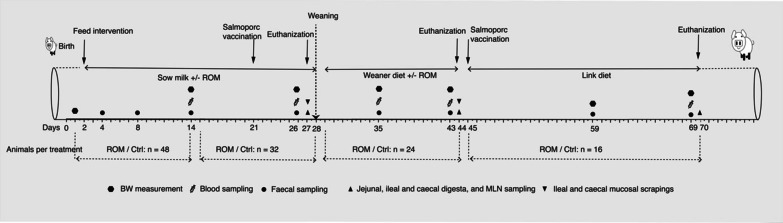
Experimental set up. Piglets orally received either ROM or tap water (Ctrl group) every other day, starting from day 2 until day 44. An oral vaccine (Salmoporc®) was given on day 21 and day 45 (booster vaccination). A randomly selected subset of animals (eight per group) were euthanised on 3 days (days 27, 44 and 70). Body weight (BW) was monitored for pig growth performance. Faeces and digesta from jejunum, ileum and caecum were collected and processed for 16S rRNA gene sequencing. Ileal and caecal mucosa were sampled for immune gene expression analysis. Blood samples were taken to assess vaccine-specific antibodies (IgM, IgA and IgG) against *Salmonella typhimurium* (Salmoporc®). Furthermore, blood samples were processed and evaluated for immune cells, and mesenteric lymph node (MLN) samples were re-stimulated with the mitogen LPS

The intervention with ROM started on day 2 and ended on day 44 (Fig. 1). The product ROM contained around 40% mycelium of *A. subrufescens,* and was provided by Selko (Trouw Nutrition, Tilburg, The Netherlands)*.* The material was freshly prepared and thoroughly mixed with tap water immediately before feeding, with a concentration of 100 mg/ml during the first week, followed by a concentration of 200 mg/ml during the remaining intervention period. During the suckling phase, piglets received ROM every other day as an oral drench (starting with 100 mg/day and doubling each week till 800 mg on day 28). An equal volume of tap water was used for the placebo (Ctrl group) using disposable syringes (Discardit II, BD). During the post-weaning phase, the dosage was increased to 1000 mg/day per administration from day 29 until day 44, after which the dietary intervention was stopped.

The study lasted 70 days starting at the birth of piglets, and eight pigs from ROM and control groups were euthanized and dissected on day 27, i.e., 1 day prior to weaning, and on days 44 and 70 (Fig. 1). During the suckling phase, all piglets were housed together with their littermates and respective biological or foster mother (pen = 5.21 m^2^ of which 0.7 m^2^ closed with tender slats in the piglet area and stainless-steel slats for the sow crate area) and adequate enrichment. The farrowing room was equipped with a computer-controlled climate system that was set to thermoneutral for the sow area, while piglets had a nesting area with floor heating and lamps. Starting 3 days before weaning (day 25), piglets had access to creep feed (weaner diet; Additional file 1: Table S1) to familiarize them with solid feed prior to weaning. On day 28, the piglets were weaned, and a subset of experimental pigs (48 pigs, 3 per pen) were randomly selected and moved to corresponding nursery pens. In all pens solid feed and water were available *ad* libitum. A weaner diet was provided from days 29 to 44, and a nursery diet from days 45 to 70 (Additional file 1: Table S1).

To assess the effect of ROM on immune function, an oral live attenuated vaccine (Salmoporc® STM (lot number 0270617), IDT Biologika GmbH, Dessau-Rosslau, Germany) of attenuated *S. Typh* was orally administered on day 21 and day 45 according to manufacturer’s instructions. The vaccine suspension was freshly prepared according to manufacturer’s instructions before each oral administration. In addition, body weight (BW) of piglets was measured at several time points (days 1, 14, 26, 35, 43, 59 and 69). The feed intake of each pen during the nursery period was also recorded, and the average daily feed intake (ADF) and feed conversion ratio (FCR) were calculated.

### Faeces, gut luminal digesta sampling and microbiota profiling

We collected faeces and luminal digesta during both the pre-weaning and post-weaning phases to evaluate the effect of ROM intervention on microbial composition. Fresh rectal faeces were sampled in cryotubes by using a wetted (with sterilized H_2_O) Puritan PurFlock Ultra cotton swab (Puritan, ME, USA) at eight time points (days 4, 8, 14, 26, 35, 43, 59 and 69) (Fig. 1) and immediately placed in a box with dry ice, followed by storage at − 80 °C until further analysis. Furthermore, at each dissection day (days 27, 44 and 70) (Fig. 1), approximately 1 g of homogenized jejunal, ileal and caecal digesta per pig were collected in a sterile cryogenic vial, snap-frozen in a box with dry ice and afterwards stored at − 80 °C until further analysis. The remainder of the digesta from each GI tract segment was mixed with sterilized H_2_O, and the pH was measured using a pH meter (ProLine). The detailed method of sample collection on each dissection day can be found elsewhere [15].

The microbial composition was analysed by sequencing of PCR-amplified and barcoded 16S ribosomal RNA (rRNA) gene fragments on the Illumina NovaSeq 6000 S2 PE150 XP platform. Faeces sampled from piglets that were dissected and all luminal digesta collected were selected for total DNA isolation with repeated bead beating for mechanical cell disruption [16]. In brief, faeces (~ 50 mg) or luminal digesta (~ 100 mg) was weighed into a screw cap tube containing sterilized 0.1 mm zirconia beads (0.25 g) and three 2.5 mm glass beads, then 300 μL Stool Transport and Recovery (STAR) buffer (Roche Diagnostics, USA) was added to the tube and followed by homogenization using a bead beater (5.5 ms, 3 × 60 s; Precellys 24, Bertin Techonologies, Montigny-le-Bretonneux, France). Afterwards samples were incubated at 95 °C with shaking at 300 rpm for 15 min, followed by centrifugation for 5 min at 16,100×*g* at 4 °C. The supernatant was collected, and the pellets obtained after centrifugation were resuspended in 200 μL STAR buffer and subjected to the same procedure as described above. The two supernatants were pooled, and 250 μL was used for the subsequent DNA purification using the automated Maxwell® 16 Research Instrument (Promega, Madison, USA) as previously described [15]. The purified DNA was eluted with 50 μL nuclease-free water (Qiagen) and the concentration was measured using a NanoDrop ND-1000 spectrophotometer (NanoDrop Technologies Inc., Wilmington, DE, USA). Purified DNA was adjusted to a concentration of 20 ng/μL and was used as a template for PCR amplification with primers 515F (5′-GTGYCAGCMGCCGCGGTAA) and 806R (5′-GGACTACHVGGGTWTCTAAT) [17], targeting the V4 region of the bacterial and archaeal 16S rRNA gene. DNA from faecal samples was amplified in triplicate in 35 μL reactions that contained 25.5 μL nuclease free water (Promega, Madison, WI, USA), 7 μL 5 × HF buffer (Thermo Fisher Scientific, Vilnius, Lithuania), 0.7 μL of 10 mM dNTPs (Thermo Fisher Scientific), 0.35 μL DNA polymerase (2 U/μL) (Thermo Fisher Scientific), 0.7 μL of 10 μM sample-specific barcode-tagged primers and 0.7 μL purified DNA (20 ng/μL) template. PCR reactions with luminal digesta DNA were performed in duplicate in a total volume of 50 μL, and the template DNA was at the same concentration (20 ng/μL) as described previously [15]. The detailed PCR program can be found elsewhere [18], and was used with minor modifications, i.e., with an annealing temperature at 50 °C for all samples, and with 30 cycles for jejunal digesta (as opposed to the 25 cycles used for other samples). Replicate PCR products were pooled and purified by using the CleanPCR kit (Clean NA, The Netherlands). Concentrations of purified DNA amplicons were determined with the Qubit BR dsDNA assay kit (Invitrogen by Thermo Fisher Scientific, Eugene, OR, USA). Finally, equimolar amounts of purified PCR products were pooled into libraries and sent for Illumina Hiseq sequencing with a read length of 2 × 150 bp (Eurofins Genomics, Ebersberg, Germany). In each library, two artificial mock communities, biological replicates of random samples and a blank (water) were included for quality control. Raw sequence data was first processed using NG-Tax 2.0 [19] with default settings and assigned to amplicon sequence variants (ASVs) using the Silva132 reference dataset [20]. ASVs with a relative abundance lower than 0.1% in a given sample were excluded on a per-sample basis.

### Gut mucosal scrapings and microfluidic qPCR for gene expression analysis

Mucosal scrapings from ileal and caecal epithelia were collected on two dissection days (days 27 and 44) (Fig. 1) to investigate the effect of ROM supplementation on pig GI tract mucosal immunity. Around 5 cm long ileal and caecal segments adjacent and proximal to the GI tract segments sampled for microbiota analysis were excised and then longitudinally cut open. Digesta were removed and segments were carefully rinsed with Phosphate Buffered Saline (PBS) without removing the mucus layer. Thereafter, the mucosa excluding the muscularis layer was removed by scraping with a scalpel, added to a snap-lock tube containing RNA later (Qiagen, Hilden, Germany), and immediately placed in a box with dry ice. Samples were shipped to the laboratory on dry ice and then stored at − 80 °C until further analysis. For RNA isolation, a 3 × 3 mm piece of tissue for each animal was cut off, added to a 2 mL Eppendorf tube containing 500 μL RLT lysis buffer (Qiagen, Hilden, Germany), and homogenized using a Turrax (IKA-Werke GmbH, Staufen, Germany) for 90 s. Subsequently, 100 μL of homogenized tissue suspension was added to a new 2 ml Eppendorf tube containing 600 μL fresh cold RLT buffer and homogenized by pipetting 10 times. Total RNA was isolated using the RNeasy Mini Kit (Qiagen, Hilden, Germany) according to manufacturer’s instructions, including on-column DNase digestion for 15 min. Qubit BR RNA Assay Kit (Thermo-Fisher, Scientific) was first used for preliminary determination of RNA concentration, degradation, and contamination. A Qsep 100 bioanalyzer (GC Biotech, Waddinxveen, The Netherlands) was then used to determine RNA quality and integrity. RNA was immediately stored at − 80 °C after isolation until cDNA synthesis. Reverse transcription was performed using the QuantiTect Reverse Transcription Kit (Qiagen, Hilden, Germany) following manufacturer’s instructions. cDNA samples were stored at − 20 °C until further use.

We selected 96 genes for microfluidic qPCR analysis based on their functional relevance to GI tract mucosal barrier function and various parts of the mucosal immune response. qPCR was performed in a BioMark HD Reader and the 96.96 Dynamic Array (Fluidigm, CA, USA). Primer pairs (n = 96) were designed using Primer3 version 0.4.0 (https://bioinfo.ut.ee/primer3-0.4.0/) for selected genes (Additional file 1: Table S2). Distribution and length of introns and exons were identified using Ensembl (https://www.ensembl.org/index.html), and interspecies-variation was checked if needed, using BLAST (https://blast.ncbi.nlm.nih.gov/Blast.cgi). Primers were synthesized at Sigma Aldrich. Pre-amplification of cDNA was performed using TaqMan PreAmp Master Mix (Applied Biosystems, Waltham, MA, United States) before qPCR. cDNA was pre-amplified in a 10 µL reaction mixture, which contained 3 µL TaqMan PreAmp Master Mix, 2.5 µL 200 nM mix of each of the 96 primer pairs, 2 µL low-EDTA TE-buffer (Panreac Applichem, Darmstadt, Germany) and 2.5 µL diluted cDNA (1:10 in low-EDTA TE-buffer). The cycling conditions were as follow: 95 °C for 10 min, followed by 19 cycles of 95 °C for 15 s and then 60 °C for 4 min. After pre-amplification, cDNA was treated with 16 U Exonuclease I (New England Biolabs, Ipswich, MA, USA) at 37 °C for 30 min, followed by 80 °C for 15 min, and then was diluted 1:10 in low-EDTA TE-buffer for microfluidic qPCR analysis. Microfluidic high-throughput qPCR was performed on a BioMark HD real-time instrument (Fluidigm) as previously described [21].

### Blood, serum, and mesenteric lymph node sampling and corresponding analysis

Blood and tissue samples were collected both pre-weaning and post-weaning to evaluate the effect of ROM intervention on the immune response. Blood samples were obtained from the jugular vein using Sodium Heparin tubes or Serum Gel tubes (S-monovette®, Sarstedt, Germany) on days 14, 26, 35, 43 and 69 (Fig. 1). Blood samples collected in Sodium Heparin tubes were used for Flow Cytometry analysis and kept at room temperature (RT) until use. Blood samples collected in Serum Gel tubes were centrifuged at 2000×g for 10 min to collect serum, which was stored at − 20 °C until use. The ileocecal Mesenteric Lymph Node (MLN) was collected after euthanasia and exsanguination on days 27, 44 and 70 (Fig. 1) and stored in ice-cold RPMI 1640 Medium (with GlutaMAX™ supplement, Gibco®) that contained 1% l-Glutamine (Gibco®) and 10% foetal calf serum (FCS, Gibco®).

Serum samples were collected to measure vaccine-specific antibodies against *S. Typh*, for which an in-house ELISA was optimized. A detailed procedure can be found elsewhere [15]. In brief, *S. Typh* bacteria were recovered from the vaccine Salmoporc®, which contains live attenuated *S. Typh*. A freshly prepared 100 μL *S. Typh* bacteria suspension (2 × 10^8^ cells/mL) was used to coat medium-binding 96 well plates (clear, flat bottom, Greiner Bio-One), and incubated overnight at 4 °C. Then, the bacterial suspension was removed, and bacteria still attached to the plate were fixed with 4% paraformaldehyde for 2 h at RT. The plates were blocked with a blocking solution consisting of 5% milk powder (ELK, FrieslandCampina, Amersfoort, The Netherlands) in demineralized water overnight at RT, and stored at 4 °C until usage. Before adding serum samples, blocked plates were washed with PBS/Tween20 (0.05%). Serum samples were diluted 250× (IgG) and 50× (IgA, IgM) in blocking solution, then 100 μL of diluted serum samples were added to the plates and incubated for 1 h at RT. After incubation, the plates were washed two times, and 100 μL of 50,000 times diluted (in blocking solution) horseradish peroxidase (HRP) conjugated goat anti-Porcine IgG, IgM, or IgA (Novus Biologicals) was added. After 30 min, plates were washed five times and incubated with 100 μL of 3,3′,5,5′-tetramethylbenzidine (TMB) substrate solution (Enhanced K-Blue®, Neogen) for 15 min. The reaction was stopped with 100 μL of 2% HCl, and the optical density (OD) of the plates was measured at a wavelength of 450 nm (Multi-Mode Microplate Reader FilterMax F5).

To measure the effects of ROM on MLN immune cells, MLN cells were restimulated with 10, 1, or 0.1 µg/mL of lipopolysaccharide (LPS, serotype O55:B5/L2880, Sigma-Aldrich) for 24 h. Next, the levels of cytokine production (IL-10) were quantified by ELISA as previously described [22]. Identification of Natural Killer (NK) cells in isolated PBMCs was performed by Flow Cytometry, followed by analysis in FlowJo™ software (Version 10) [15]. Activated NK cells were identified as CD3^+^CD8α^+^CD25^+^. The gating strategy for NK cell identification was designed according to previous studies [23–25].

### Statistical analysis

For the growth performance data, BW was analysed with a linear mixed model for repeated measures analyses.

For microbiota data, ASV read counts were first transformed to relative abundance, and all statistical analyses were performed in R 3.6.1 [26]. Alpha diversity, with metrics of observed species, phylogenetic diversity, Shannon diversity and inverse Simpson (InvSimpson), was determined at ASV level using packages *picante* [27] and *microbiome* [28], followed by a Linear Mixed-Effects Model that was applied to assess whether alpha diversity was significantly different between ROM and Ctrl piglets over time. Beta diversity was estimated at ASV level based on weighted UniFrac [29] and unweighted UniFrac distance [30], and the results were further plotted by unconstrained principal coordinate analysis (PCoA) using the *phyloseq* R package [31]. Both metrics take the phylogenetic relationships among ASVs into account, with unweighted UniFrac only considering the presence or absence of ASVs, whereas weighted UniFrac takes the relative abundance of ASVs into account. The significance of differences in beta diversity was computed by permutational multivariate analysis of variance (PERMANOVA), using the Adonis’ function in the *vegan* package [32]. If there were significant differences in overall microbial composition between two groups, the linear discriminant analysis effect size (LEfSe) method [33] was used to identify biomarkers characterizing differences between ROM and Ctrl groups. A nonparametric Wilcoxon rank sum test was applied to assess whether the inter-individual variation of weighted and unweighted UniFrac distances was significantly different between both groups at each time point. In order to test for differences in relative abundance of microbiota at genus level between ROM and Ctrl groups over time, a generalized additive model for location, scale, and shape with a zero-inflated beta family (GAMLSS-BEZI) was used, as implemented with the *taxa.compare* function in the *metamicrobiomeR* package [34]. Significance was assessed with a false discovery rate correction for all multiple testing according to the procedure by Benjamini–Hochberg, with a threshold of 0.05 [35]. Differences were considered significant if adjusted *p* ≤ 0.05, or as a trend if the adjusted *p* value was below 0.1 but above 0.05 (0.05 < *p* ≤ 0.10).

For microfluidic qPCR data of gut mucosal gene expression, the method for data pre-processing was as described previously [36]. In brief, data was first pre-processed, normalized and relatively quantified per time point and tissue type, using GeneEx5 (MultiD, Göteborg, Sweden). The algorithms geNorm [37] and NormFinder [38] were then used for reference gene selection per tissue origin (ileum or caecum). Primer pairs that resulted in inconsistent replicates were excluded from the dataset. The Cq values were converted to relative quantities for each primer assay, and relative quantities were Log2-transformed before downstream statistical analysis. Redundancy analysis (RDA) was performed to assess multivariate effects of environmental variables (treatment, time, pH and BW) on ileal and caecal mucosa gene expression, as implemented by the capscale function in the *vegan* package [32]. Missing values of pH and BW were estimated using the K-nearest neighbour algorithm from the *vim* package [39]. The statistical significance was estimated by an ANOVA-like permutation test with 999 permutations using the anova.cca function in the *vegan* package [32]. Differential gene expression between ROM and Ctrl groups per tissue and time point was tested using a linear model framework with an empirical Bayes moderated t-test, and resulting *p* values were adjusted for multiple testing using the Benjamini–Hochberg false discovery rate (FDR) from the *limma* R package [40]. − log10 adjusted *p* value versus log2 fold-change in expression for each pairwise comparison was plotted using *EnhancedVolcano* R package [41]. Differences were expressed as significantly down- or up-regulated if adjusted *p* ≤ 0.05 with a log_2_ fold change > 0.5 or tendencies if 0.05 < *p* ≤ 0.1 with a log_2_ fold change > 0.5.

To determine the strength of association between gut mucosal gene expression and corresponding relative abundance of microbial groups at genus level, the microbial composition data was filtered with relative abundance and prevalence thresholds of > 0.1% and 40% of the samples, respectively. Furthermore, gene expression data was log transformed before correlation analysis that was performed according to Pearson’s product moment (95% CI), and the reported *p* values were corrected for multiple comparisons according to the Benjamini–Hochberg method.

For immunological analyses, a Linear Mixed Model was used to assess the immunological effects over time and the interaction between time and treatment, using R statistical software (version 3.6.2). Additionally, unpaired Student’s t-tests were performed to assess the differences between the treatment groups per time point. Normality of data (Shapiro–Wilk test) and homogeneity of variances (Levene’s test) were checked prior to statistical testing. Skewness values between − 2 to + 2 were considered acceptable [42]. Extreme outliers (indicated by R) were removed from the analysis. When the normality was not met, data were log-transformed, but presented as untransformed means. Results with an adjusted *p* value below 0.05 were considered statistically significant and results between 0.05 and 0.1 were considered a trend.

## Results

### Growth performance

The dynamic effects of oral ROM supplementation for pigs in early life on BW, ADF and FCR are displayed in Additional file 1: Fig. S1. Overall, no statistical differences on BW, ADF and FCR between Ctrl and ROM groups were observed. However, a tendency (*p* = 0.08) on FCR was observed from day 28 to 35, i.e. the first week after weaning, where ROM pigs showed a lower level of FCR than Ctrl group.

### Effect of ROM supplementation on pig gut microbiota development

**Fig. 2:**
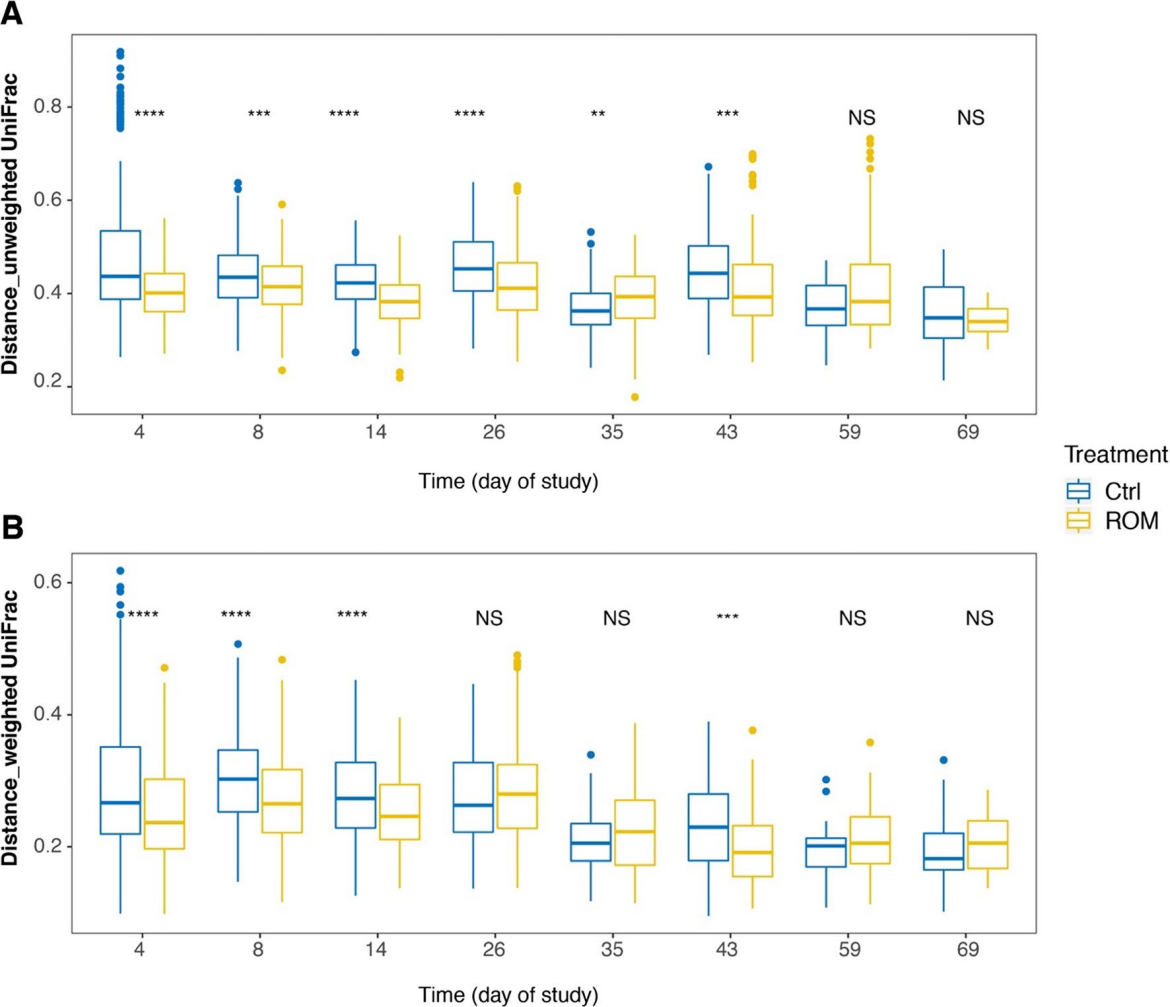
The effect of ROM treatment on faecal microbial inter-individual variation. Boxplots show the inter-individual variation for ROM versus Ctrl pigs in faecal microbial composition over time based on unweighted (**A**) and weighted (**B**) UniFrac distances. Significance between treatments was assessed by Wilcoxon rank sum test per time point. Asterisks are used to indicate the statistical differences between treatments (**p < 0.01, ***p < 0.001, ****p < 0.0001). Blue and yellow colours represent control (Ctrl) and treated (ROM) groups, respectively

**Fig. 3:**
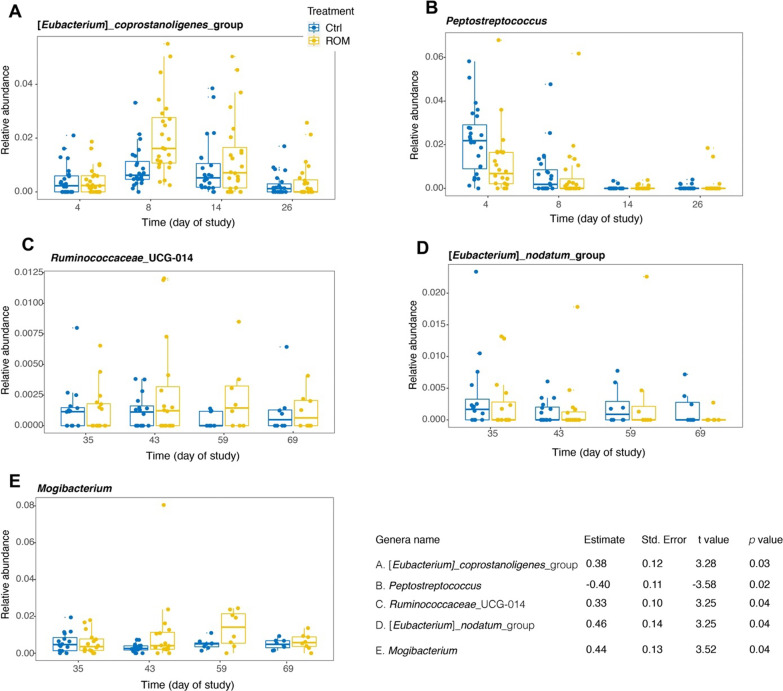
Differentially abundant genera between ROM and Ctrl pigs during pre-or post weaning. Plotted genera were identified through GAMLSS-BEZI model with random effect. The *p* value was corrected by FDR for multiple testing. Blue and yellow colours represent control (Ctrl) and treated (ROM) groups, respectively

To evaluate the potential effect of ROM supplementation on gut microbiota development, microbial composition was determined for all faecal samples from suckling and nursery phases at eight time points (days 4, 8, 14, 26, 35, 43, 59 and 69), as well as jejunal, ileal and caecal luminal digesta at each dissection day (days 27, 44 and 70). As expected, time (i.e., age of piglets) together with the diet switch from milk to solid feed were the two major drivers for faecal microbiota development, both with respect to alpha- as well as beta- diversity (Additional file 1: Figs. S2 and S3). No significant differences were observed between treatments either on faecal microbial alpha- or beta-diversity. However, the alpha diversity with indices of observed richness and InvSimpson tended to be higher in Ctrl piglets than ROM groups during pre-weaning (Additional file 1: Fig. S2A, D). The inter-individual variation of faecal microbiota was significantly higher in the Ctrl group than in ROM piglets based on unweighted UniFrac distances during pre-weaning and on day 43 (Fig. 2A). Similarly, based on weighted UniFrac distances, Ctrl animals had higher inter-individual variation than ROM piglets during the same period except for day 26 (Fig. 2B). The inter-individual variation was significantly larger in ROM piglets than Ctrl groups on day 35 based on unweighted UniFrac distance, but this was not observed when using weighted UniFrac distance. In terms of the relative abundance of faecal microbial taxa at genus level, GAMLSS-BEZI model analysis identified that ROM pigs had higher relative abundance of [*Eubacterium*]_*coprostanoligenes*_group, but lower relative abundance of *Peptostreptococcus* than Ctrl animals before weaning (Fig. 3A, B). Furthermore, ROM pigs tended to have lower relative abundance of *Alloprevotella*, *Bilophila* and *Christensenellaceae*_R-7_group, but higher relative abundance of *Ruminococcaceae_*UGG-002 and *Blautia* than Ctrl pigs before weaning (Additional file 1: Fig. S4A–E). During post-weaning (i.e., from day 35 to day 69), we found ROM pigs had higher level of *Ruminococcaceae*_UGG_014 and *Mogibacterium*, but lower level of [*Eubacterium*]_*nodatum*_group than Ctrl animals (Fig. 3C–E). Moreover, *Succinivibrio* was found to show a tendency towards higher relative abundance in ROM pigs compared to Ctrl animals (Additional file 1: Fig. S4F).

**Fig. 4:**
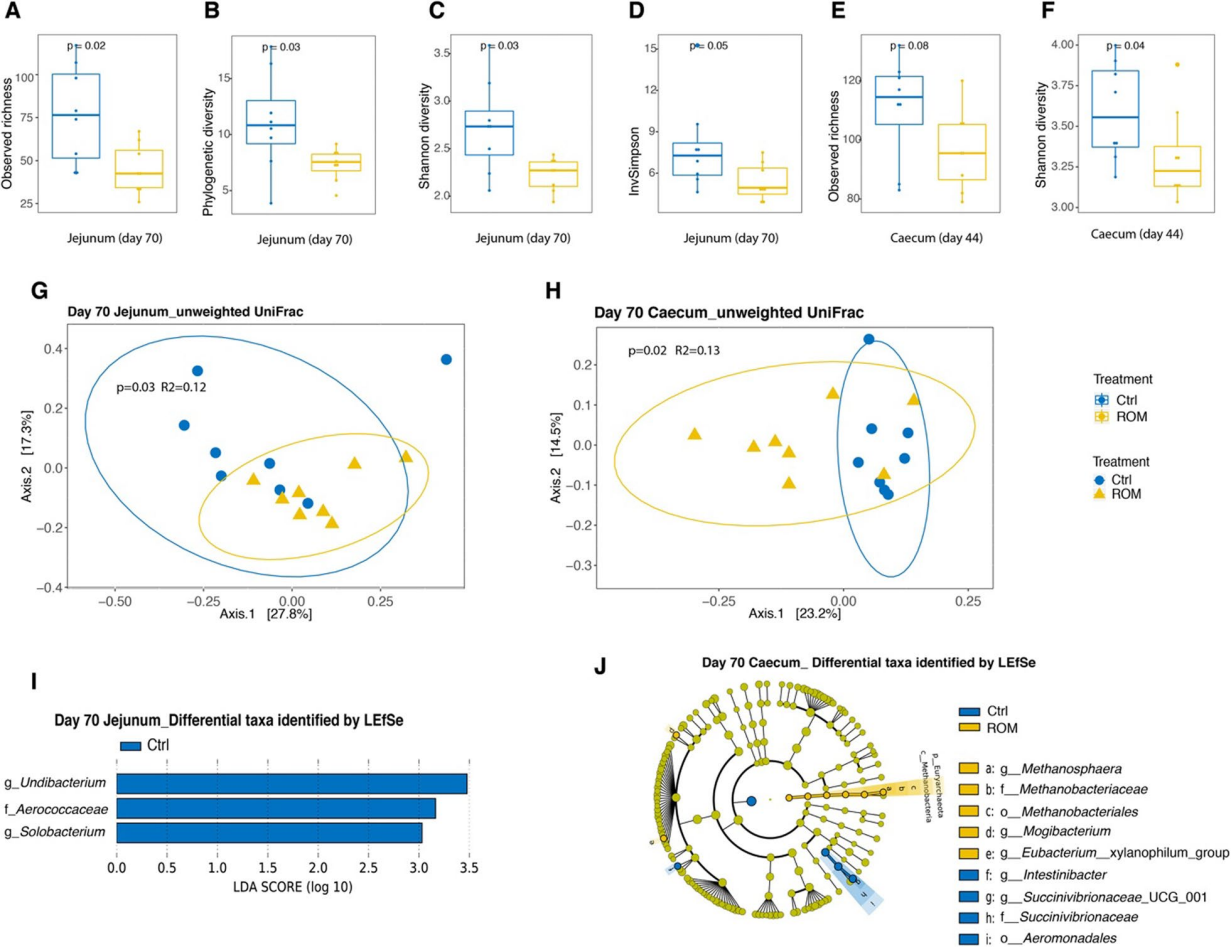
The effect of ROM treatment on luminal microbiota colonization. Boxplots show comparisons of jejunal luminal microbial alpha diversity between ROM and Ctrl groups on day 70 based on observed richness (**A**), phylogenetic diversity (**B**), Shannon diversity (**C**) and inverse Simpson (InvSimpson) (**D**), as well as caecal luminal microbial diversity between two groups on day 44 with indices of observed richness (**E**) and Shannon diversity (**F**). Differences in alpha diversity between groups were evaluated by nonparametric Wilcoxon rank sum test. Principal coordinate analysis (PCoA) plots for jejunal luminal microbiota (**G**) and caecal luminal microbiota (**H**) at ASV level both on day 70 based on unweighted UniFrac. Significance of the difference between ROM and Ctrl groups was assessed using PERMANOVA. The percentages at the axes indicate the variation explained. Linear discriminant analysis (LDA) Effect size (LEfSe) of differentially abundant jejunal taxa between ROM and Ctrl groups on day 70 (**I**). Cladogram of caecal differentially abundant taxa between ROM and Ctrl pigs on day 70 according to LEfSe (**J**). Blue and yellow colours represent control (Ctrl) and treated (ROM) groups, respectively

Time post parturition was also a major factor linked to luminal microbiota composition and diversity, irrespective of the diversity metrics used (Additional file 1: Figs. S5 and S6). When all samples from three time points were included for the analysis per intestinal location, administration of ROM had a significant effect on jejunal luminal microbial diversity, with lower levels of phylogenetic diversity than in Ctrl piglets (Additional file 1: Fig. S5B). Furthermore, significant time × treatment interactions were found for jejunal and caecal alpha diversity, using observed richness and phylogenetic diversity, respectively (Additional file 1: Fig. S5A, J). Post hoc comparison found ROM pigs had significantly lower levels of observed richness of jejunal microbiota than Ctrl pigs on day 70. When luminal digesta samples at each location were separated for analysis according to time, ROM pigs showed lower jejunal alpha diversity than Ctrl animals on day 70, irrespective of the used metrics (Fig. 4A–D). Similarly, ROM pigs tended to have a lower value of observed species and had lower Shannon diversity than Ctrl pigs (Fig. 4E, F). No treatment effect was observed on luminal microbial beta diversity for any intestinal location irrespective of the used metric, i.e., unweighted UniFrac or weighted UniFrac distance, when all time points were analysed together (Additional file 1: Fig. S6A–E). When time points were analysed separately, PCoA showed significant differences in jejunal and caecal microbial composition between treatments based on unweighted UniFrac on day 70 (Fig. 4G, H). The LEfSe algorithm was then used to identify differentially abundant taxa that were most strongly associated with the observed difference on day 70. This revealed that the genera *Undibacterium* and *Solobacterium* and the family *Aerococcaceae* were only present in Ctrl pigs in the jejunum (Fig. 4I and Additional file 1: Fig. S7A–C). For the caecum, two genera, including *Mogibacterium* and *Eubacterium*__*xylanophilum*_group, were more abundant in ROM pigs, whereas ROM pigs had lower relative abundance of genera *Intestinibacter* and *Succinivibrionaceae*_UCG_001 (Fig. 4J). At higher taxonomic level, the bacterial family *Succinivibrionaceae* and the order *Aeromonadales* were less abundant in ROM pigs (Fig. 4J). Intriguingly, archaeal members of the genus *Methanosphaera*, *Methanobacteriaceae* at family level and *Methanobacteriales* at order level were only present in ROM pigs (Fig. 4I and Additional file 1: Fig. S7D–F).

### The effect of ROM intervention on mucosal gene expression

**Fig. 5:**
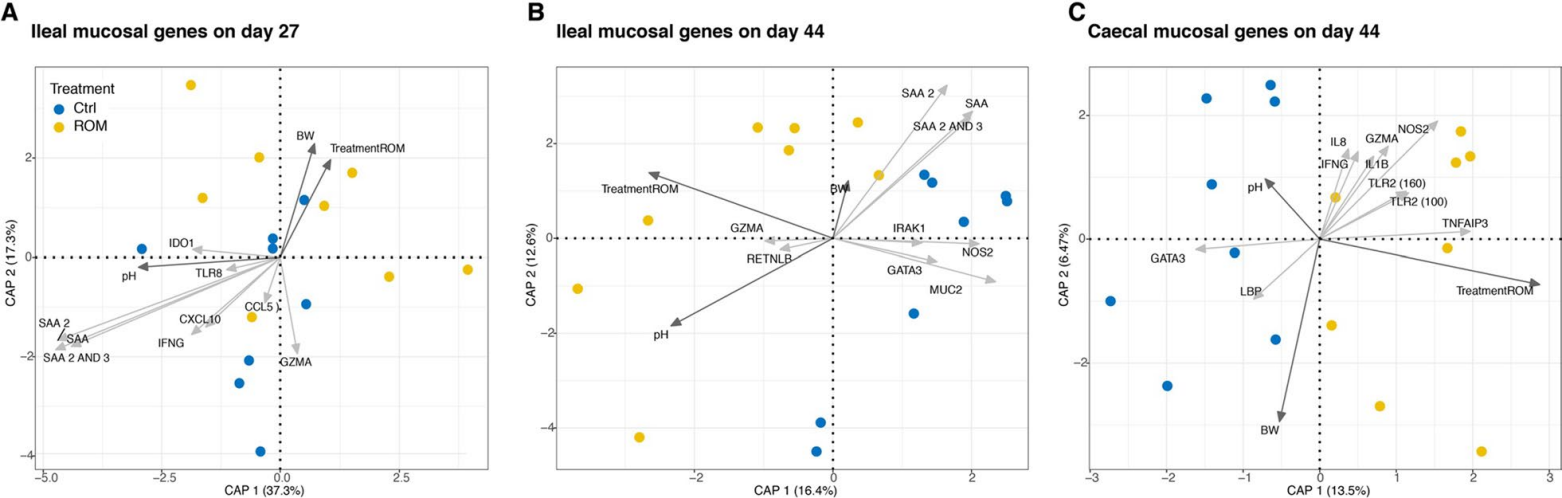
Redundancy analysis (RDA) triplots show the association between the variation of gene expression and environmental variables, focusing on samples from ileum on day 27 (**A**) and day 44 (**B**), as well as caecum (**C**) on day 44. Blue and yellow colours represent control (Ctrl) and treated (ROM) groups, respectively. Dark grey arrows indicate environmental variables and light grey arrows show genes for which variation in expression levels is best explained in the model. The percentages at the axes indicate the variation explained by the first two canonical axes

To investigate the effect of the ROM intervention on mucosal immunity we performed high-throughput microfluidic qPCR for 96 genes using ileum and caecum tissue from day 27 (1 day pre-weaning) and day 44 (16 days post-weaning). Of the 96 selected genes, 78 were considered expressed in the ileum and 79 in the caecum. Redundancy analysis (RDA) revealed a strong effect of sampling day in both tissues (Additional file 1: Fig. S8A, B). Other variables such as treatment (*p* = 0.009) and pH (*p* = 0.04) also significantly contributed to explaining the observed variation in ileal gene expression, and significant interactions were found between BW × time (*p* = 0.02) and pH × time (*p* = 0.001). When we analysed ileal gene expression data separately for the different sampling days, the variables treatment (*p* = 0.05), ileal pH (*p* = 0.001) and BW (*p* = 0.02) all contributed to explaining the observed variation in ileal mucosal gene expression on day 27 (Fig. 5A), whereas treatment (*p* = 0.04) and ileal pH (*p* = 0.03) significantly contributed to explaining the variation of ileal mucosal gene expression on day 44 (Fig. 5B). For caecal mucosal gene expression data, only treatment significantly explained the variation in gene expression on day 44 (*p* = 0.05) (Fig. 5C), whereas none of the variables significantly explained the variation on day 27 (Additional file 1: Fig. S8C).

In the ileum, expression of several immune genes (e.g., IFNG, CXCL10/IP10) as well as serum amyloid A genes (SAA1, SAA2 and SAA3), all of which are known to be induced by inflammatory stimuli, was found to be negatively associated with ROM treatment (Fig. 5A, B). In contrast, expression of several immune genes[(e.g., IFNG, IL8 and toll-like receptor 2 (TLR2)] was positively associated with ROM treatment in the caecum on day 44 (Fig. 5C). These findings indicate differences in the effect of ROM supplementation on ileal and caecal mucosa.

**Fig. 6:**
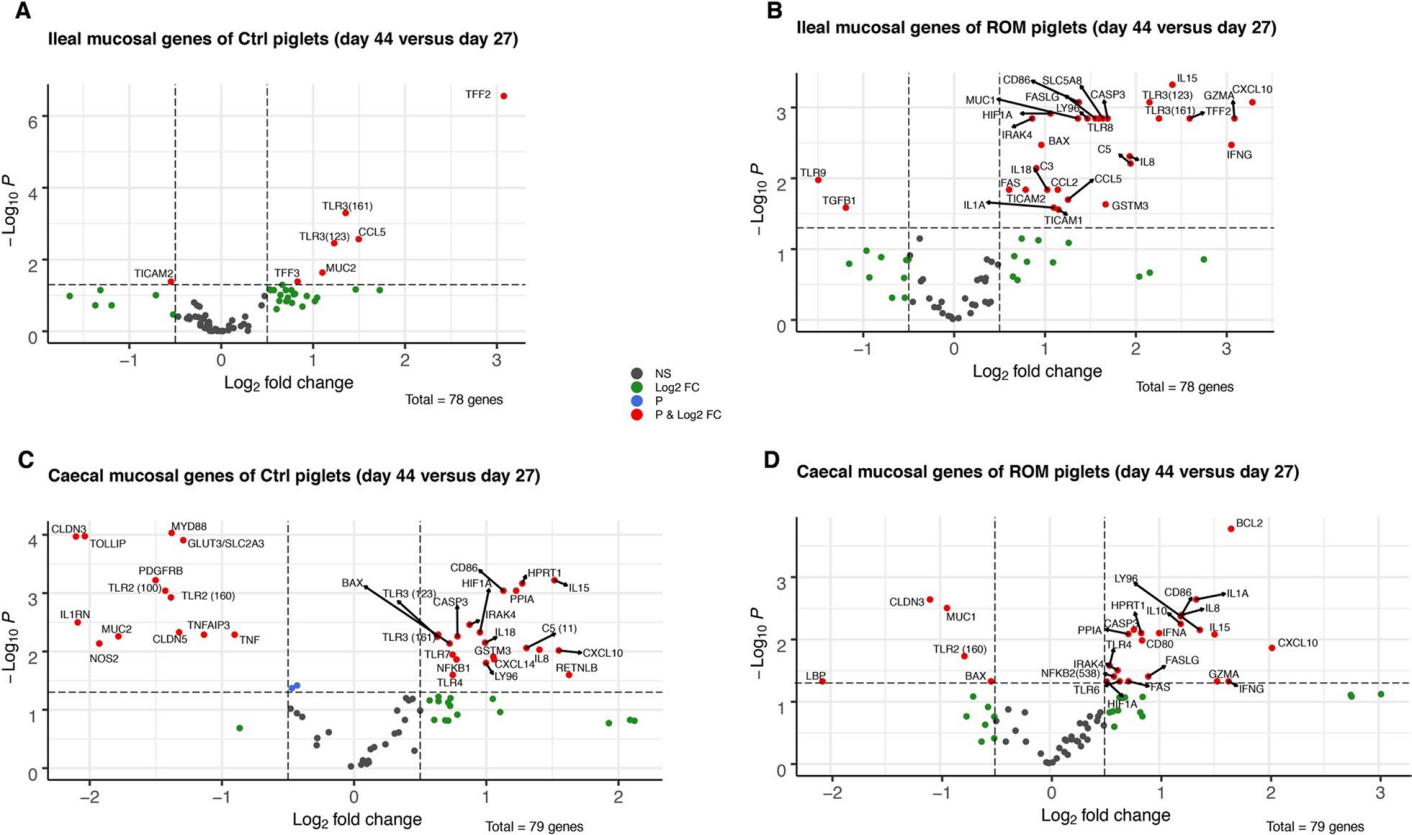
Volcano plots of all expressed genes in mucosa collected from ileum of Ctrl- (**A**) and ROM (**B**) piglets, as well as from caecum of Ctrl- (**C**) and ROM (**D**) piglets by comparisons between day 44 and day 27 within each group. The x-axis is the log2 fold change (log2 FC) for the ratio day 44 versus day 27 and the y-axis is the − log10 *p* value that was adjusted by FDR for multiple testing. The red dots represent genes that were either significantly more (positive fold-change) or less (negative fold-change) expressed on day 44 versus day 27 for the respective location and experimental group, with adjusted *p* ≤ 0.05 and a log2 FC > 0.5. Blue points indicate differentially expressed genes with an adjusted *p* < 0.05 and a log2 FC < 0.5. Green points indicate differentially expressed genes with adjusted *p* > 0.05 and a log2 FC > 0.5

Analysis of differentially expressed genes (DEG) on day 44 versus day 27, showed several ileal and caecal mucosal genes were differentially expressed post-weaning in both Ctrl and ROM-treated groups compared to pre-weaning (Fig. 6A–D). A larger number of genes were differentially expressed in ileum on day 44 compared to day 27 in the ROM group compared to Ctrl animals (Fig. 6A, B). In the caecum this was opposite with fewer differentially expressed genes on day 44 compared to day 27 in the ROM-treatment group than in Ctrl animals. (Fig. 6C, D).

In ileal mucosa of Ctrl pigs three of the five DEGs with increased expression on day 44 were MUC2 and TFF2 and TFF3, which are expressed in goblet cells and involved in epithelial barrier function and restitution. Additionally, CCL5 and TLR3 were upregulated in the ileum of the Ctrl group after weaning on day 44. In the ileum of ROM treated pigs several genes involved in TLR signalling (including TICAM1, TICAM2, IRAK4, TLR3 and TLR8, LY96), inflammatory responses (IFNG, IL18, IL8, C3, C5, CXCL10, CCL2, IL1A and GZMA), redox signalling (HIF1A and GSTM3) and apoptosis pathways (FASLGF, FAS, BAX, CASP3) showed higher expression levels on day 44 as compared to day 27. In addition, the membrane bound mucin encoding gene MUC1 and the short-chain fatty acid transporter encoding gene SLC5A8 were upregulated in the ileum of ROM treated pigs post weaning.

In the caecal mucosa of Ctrl pigs, weaning (day 44 vs. day 27) led to a decrease in expression of MUC2, the glucose transporter encoding gene GLUT3, genes involved in TLR signalling MyD88, genes negatively regulating inflammatory signalling (TOLLIP, TNFAIP3 and ILRN), genes encoding the epithelial tight junction claudins (CLDN3 and CLDN5), genes related to redox balance (NOS_2_, PDGFRB) as well as TNF. DEGs which were upregulated in the caecum of Ctrl pigs when comparing day 44 and day 27 included genes involved in TLR signalling (TLR3, TLR7, NFKB1), encoding cytokines and chemokines (IL-18, C5, CXCL14) as well as BAX and RETNLB. In the caecum of ROM treated pigs, genes encoding membrane associated mucin MUC1, LPS binding protein (LBP) and BAX were down-regulated between day 44 and 27. Two genes related to TLR signalling (TLR6, NFKB2), cytokines (IL1A, IFNA, IL-10, GZMA,) and genes involved in apoptosis pathways (FAS, CASP3, FASLG, BCL2) were upregulated after weaning in the cecum of the ROM treated group.

**Fig. 7:**
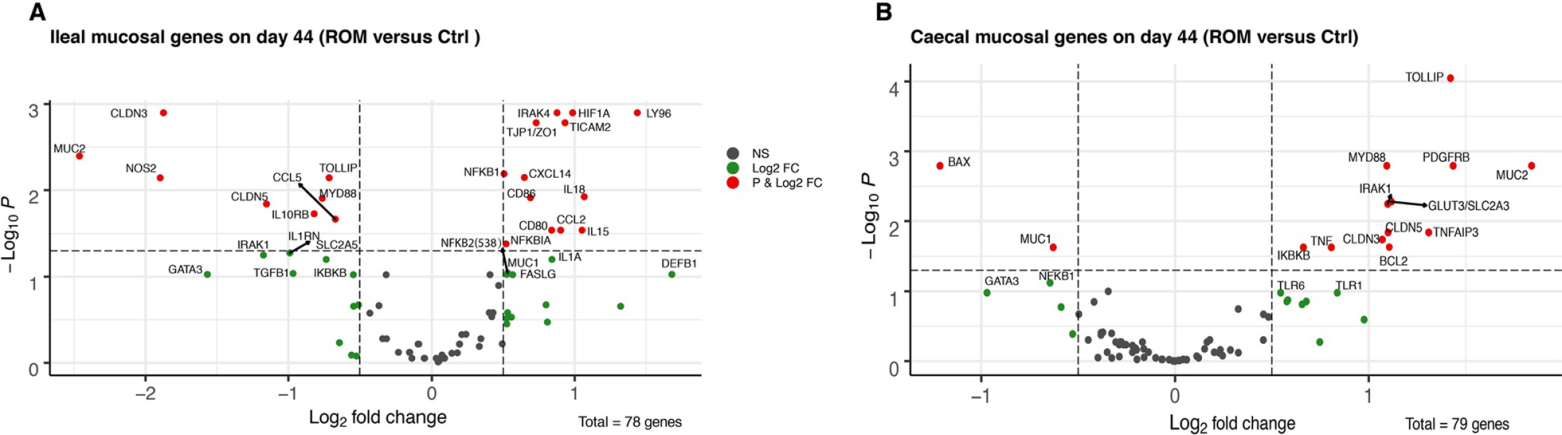
Volcano plots of genes expressed in ileal (**A**) and caecal (**B**) mucosa on day 44, respectively. The x-axis is the log2 fold change (log2 FC) for the ratio ROM treatment versus Ctrl group and the y-axis is the − log10 *p* value that was adjusted by “FDR” for multiple testing. The red dots represent genes that were either significantly more (positive fold-change) or less (negative fold-change) expressed in the ROM group as compared to the Ctrl group, with adjusted *p* ≤ 0.05 and a log2 FC > 0.5. Green points indicate differentially expressed genes with adjusted *p* > 0.05 and a log2 FC > 0.5

Genes that were differentially expressed in the ROM versus Ctrl groups were only identified on day 44 (Fig. 7). In the ileum, ROM treatment increased expression of genes encoding tight junction protein TJP1/ZO1 but decreased expression of genes encoding claudins (CLDN3 and CLDN5) and MUC2 compared to Ctrl pigs. In contrast MUC2, CLDN3 and CLDN5 were strongly upregulated in the caecum of ROM supplemented pigs. Among genes involved in TLR signalling, we observed increased expression of NFKBIA, TICAM2, NFKB1, IRAK4, LY96 and decreased expression of MYD88 and the inhibitory TLR adaptor protein TOLLIP, in the ileal mucosa of ROM treated pigs compared to Ctrl pigs. In the ROM group expression of genes encoding cytokines IL18, CXCL14, IL15, CCL2, and myeloid associated receptors CD80 and CD86 was increased in the ileum, whereas expression of CCL5 was decreased compared to Ctrl pigs. The expression of the two genes NOS2 and HIF1A related to redox signalling in the ileal mucosa of ROM pigs compared to Ctrl animals was decreased and increased, respectively.

In the caecum, TNF was the only cytokine-encoding gene with increased expression in ROM animals compared to Ctrl pigs on day 44. MUC2, IRAK1, IKBKB, PDGFRB, GLUT3, BCL2, claudins CLDN5 and CLDN3 and the negative regulators of inflammatory signalling (TOLLIP, TNFAIP3) were more highly expressed in the caecum of the ROM group than the Ctrl group, whereas the expression levels of BAX and MUC1 were lower in caecal mucosa of ROM animals than in Ctrl pigs.

### Association between the relative abundance of intestinal luminal genera and corresponding mucosal gene expression

Correlation analysis was performed to investigate the association between predominant intestinal luminal genera with corresponding mucosal gene expressed levels in the ileum and caecum mucosa on day 27 and 44. In the ileum, correlation analysis revealed only the genus *Streptococcus* was negative correlated with the gene NFKB1 on day 27 (Additional file 1: Fig. S9). On day 44, two genera were found to be negatively correlated with some ileal genes, where *Clostridium_*sensu_stricto_6 was negatively correlated with genes SAA 2, SAA and SAA 2 AND 3, and *Romboutsia* was negatively correlated with TFF3 (Additional file 1: Fig. S10). *Clostridium_*sensu_stricto_1 was negatively associated with gene C3 but positively correlated with TLR1 (Additional file 1: Fig. S10). Correlation analysis also revealed some correlation between caecal microbiota relative abundance and mucosal gene expression levels on day 27 and day 44, respectively. On day 27, the relative abundance of genera Family_XIII_AD3011_group, *Alloprevotella*, and [*Eubacterium*]_*coprostanoligenes*_group were negatively correlated with the gene expression levels of TLR3 (123), TICAM2 and IRAK4, respectively (Additional file 1: Fig. S11). On day 44, two genera, i.e., unclassified genus from *Veillonellaceae*_and *Rikennellaceae*_RC9_gut_group were negatively correlated with the genes C5 and BCL2, respectively (Additional file 1: Fig. S12), while unclassified genus and uncultured_bacterium of *Muribaculaceae* were positively correlated with TP53 (494) and TFF3, respectively (Additional file 1: Fig. S12).

### The effect of ROM supplementation on vaccination response, ex vivo stimulation of MLN cells and the activation of natural killer cells

**Fig. 8:**
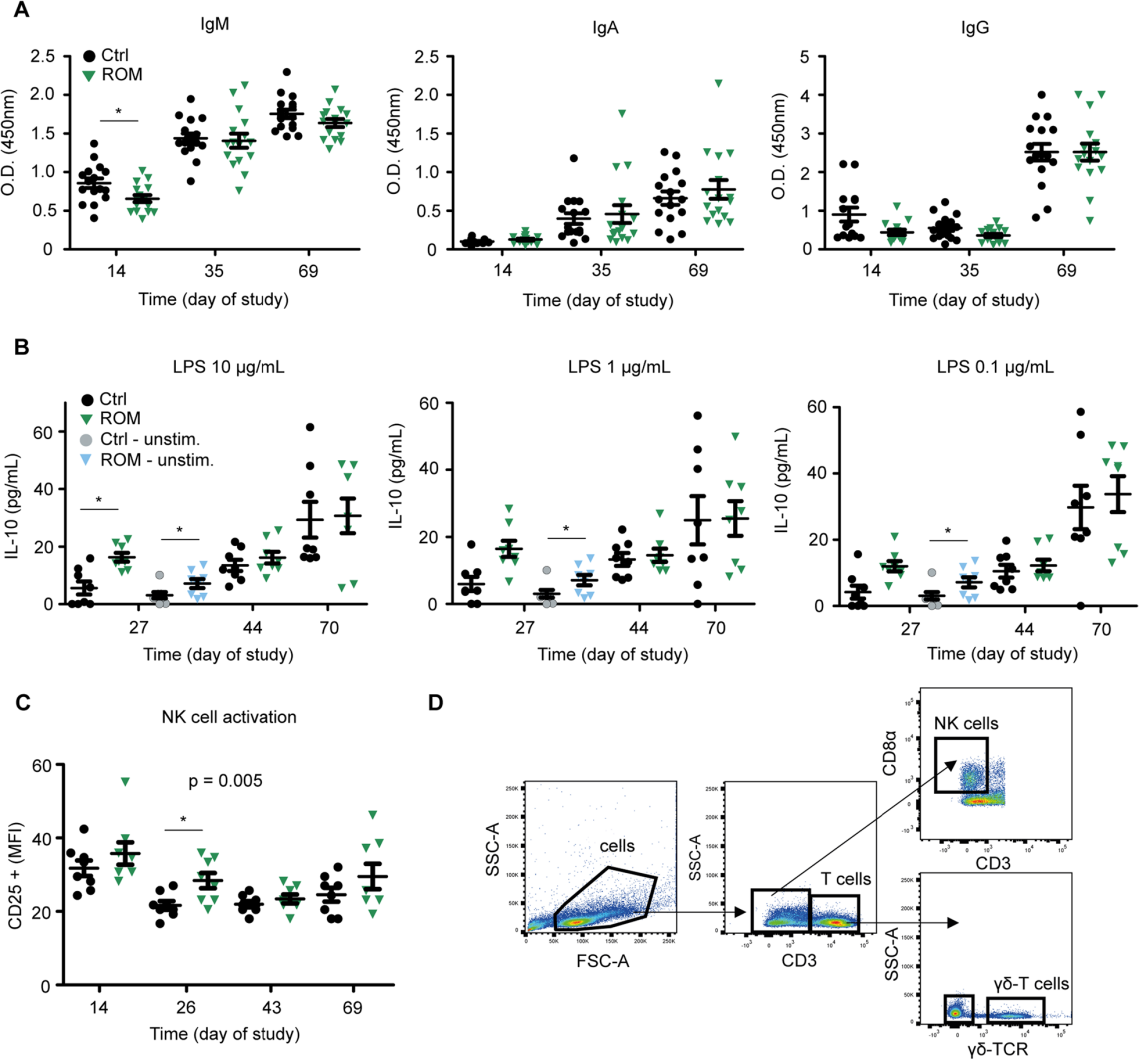
Immunological analysis. **A** Serum levels of *Salmonella*-specific IgM, IgA, and IgG prior to vaccination (day 14), 2 weeks after the initial vaccination (day 35), and 3 weeks after the booster vaccination (day 69). **B** IL-10 production upon ex vivo stimulation of MLN cells. MLN cells were stimulated for 24 h with 10, 1, and 0.1 μg/mL of LPS, or left unstimulated (cell culture medium only). **C** Activation of Natural Killer cells in PBMCs. **D** Gating strategy to identify Natural Killer cells. *p* values indicate significant differences between treatment groups over time, and asterisks are used to indicate significant differences between treatment groups after taking into account single time points (*p < 0.05). Every point represents an individual animal from a separate pen (n = 7 or 8 per treatment group), and error bars represent standard deviations. Normal distribution and equal variances of data were checked and log-transformed when necessary. No differences in IL-10 production by unstimulated MLN cells were observed post-weaning and are therefore not presented

To measure if ROM influences the competence of the immune system, an oral vaccination against *S. Typh* was administered on day 21 and day 45. As expected, a clear and significant increase of serum *Salmonella*-specific IgM and IgA antibodies was observed after both the primary and the secondary (booster) vaccination (Fig. 8). An increase in antigen-specific IgG antibodies was only observed after the second (booster) vaccination (Fig. 8A). Despite the clear increase of antibody titres over time, there were no significant differences in the amount of *Salmonella-*specific IgA and IgG between the treatment groups, pre- or post-weaning. Interestingly, ROM-treated animals showed a significant decrease (*p* = 0.025) of *Salmonella*-specific IgM when including all time points. Furthermore, MLN cells from ROM-treated animals produced higher levels of IL-10 upon stimulation with 10 µg/mL LPS (*p* = 0.013; T-test) in comparison to LPS stimulated MLN cells from Ctrl animals (Fig. 8B). Interestingly, MLN cells from ROM-treated animals also produced more IL-10 when left unstimulated (medium only) compared to unstimulated MLN cells from the Ctrl animals (*p* = 0.016; T-test). No such differences were observed for the post-weaning time points (day 44 and day 70, respectively). Moreover, NK cells from ROM-treated animals were more activated than NK cells from Ctrl animals, considering all time points (*p* = 0.005). As for single time point analysis, a significant difference between the treatment groups was only observed on day 26 pre-weaning (*p* = 0.017) (Fig. 8C).

## Discussion

Using feed additives as alternatives for antibiotics to shape gut functioning and host immune competence has received an increasing interest for piglets in suckling and nursery phases [43, 44]. However, results obtained so far from many feed additives lack consistency, and their efficacy seems to be influenced by a variety of external factors (i.e., health status, diet formulation and farms) [45, 46]. Hence, more research is required to assess the effects of novel feed additives on GI tract health and development, as well as on the host’s immune competence in early life. To the best of our knowledge, this is the first interdisciplinary study to assess the impact of a fungi fermented rye feed additive (ROM) on pig gut microbiota composition, intestinal mucosal and systemic immunity development during suckling-to-nursery phases.

As hypothesised, ROM supplementation affected the development of piglet gut microbiota, but the modulation was modest. Pigs supplemented with ROM tended to have a lower alpha diversity of faecal microbiota compared to Ctrl piglets during pre-weaning, as based on observed richness and InvSimpson. These alpha diversity indices emphasise on taxa richness and evenness [47, 48], respectively. Our findings are consistent with other publications, where supplementation with different fibres also induced lower levels of microbial alpha diversity [49, 50]. In general, a higher alpha diversity often contributes to ecosystem stability, resilience and gut health [51]. However, the reduced alpha diversity observed here may have been a result of the selection of taxa stimulated by the functional compounds in ROM, and not necessarily indicate decreased microbiota stability. This is supported by the observation of a significantly lower inter-individual variation of faecal microbiota among ROM piglets compared to Ctrl animals during the pre-weaning phase. To more specifically assess whether ROM supplementation modulated gut microbiota development at genus level over time, the GAMLSS-BEZI model identified that ROM treated piglets had a significantly higher relative abundance of [*Eubacterium*]_*coprostanoligenes*_group, whereas *Peptostreptococcus* was significantly more abundant in Ctrl piglets during pre-weaning. The abundance of [*Eubacterium*]*_coprostanoligenes*_group has previously been reported to positively respond to dietary *Bacillus subtilis* supplementation for piglets around weaning [52], and be negatively associated with diarrhoea incidence of piglets [53]. In contrast, the genus *Peptostreptococcus* may include potential porcine intestinal pathogens and was associated with the level of *Clostridioides difficile* in suckling piglets [54]. Intriguingly, significant differences on beta diversity of jejunal and caecal luminal microbiota on day 70 were observed between ROM and Ctrl pigs using unweighted UniFrac distance, even though the diet intervention ceased on day 44. LEfSe analysis further revealed that two genera belonging to the *Proteobacteria,* including *Undibacterium* and *Solobacterium*, were only present in Ctrl pigs in the jejunum on day 70, and two other proteobacterial genera (*Intestinibacter* and *Succinivibrionaceae*_UCG_001) were less abundant in ROM pigs in the caecum on day 70. This reduction in members of *Proteobacteria* in ROM pigs in jejunum and caecum on day 70 may be considered beneficial as the increased prevalence of this phylum is a potential signature of intestinal dysbiosis [55]. Conversely, the archaeal genus *Methanosphaera* was only present in ROM pigs in the caecum on day 70. A higher abundance of *Methanosphaera* spp. in pigs has been reported to be associated with fat metabolism [56], however, metabolic parameters were not measured in the present study. It could be speculated that observed differences in luminal microbiota observed on day 70 may be due to altered host immune parameters caused by ROM supplementation from day 2 up to day 44 as the piglets were exposed to the same diet and housing conditions afterwards.

The intestinal mucus layer is one of the first lines of intestinal protection. Mucins expressed by epithelial cells are the major structural and functional components of mucus [57–59]. In the current study, mucins MUC1 and MUC2 were differentially expressed at both ileal and caecal mucosa in response to weaning, albeit not consistently for ROM and Ctrl pigs. In the intestine, MUC1 is one of the transmembrane mucins that have been implicated in cell signalling and immune modulation, while MUC2 is the predominant gel-forming mucin contributing to the formation of the mucus barrier and preventing the adhesion and colonization of pathogenic microorganisms [60]. The modulation of MUC1 by ROM supplementation may be involved in cell signalling of immune responses during the weaning transition. It should be noted, however, that the observed differences in expression of MUC1 between ROM and Ctrl pigs were relatively minor for both ileum and caecum on day 44, which might limit biological impact on pig health. The lower MUC2 expression in ileum and higher expression in caecum on day 44 compared to Ctrl pigs is in line with a previous report by Palamidi et al. [61], where diet supplementation with an organic acids-based formulation for broilers also resulted in decreased expression of MUC2 in the ileum but an increased caecal expression in comparison with a control group, and without sign of abnormal inflammation. It is known that MUC2 secretion along the intestine can be initiated by the activation of signalling pathways targeting transcription factors (e.g., NF-κB), and different bioactive factors, such as gut microbes, microbial metabolites and cytokines, are involved in mediation of MUC2 transcription [62]. Our findings suggest that different mechanisms may be underlying the regulation of MUC2 secretion in the pig ileum and caecum.

Tight junctions (TJs) play a critical role in regulating the permeability of the intestinal barrier [63]. Weaning did not affect the expression of TJ proteins in the ileum in both groups, but ROM supplemented pigs had lower levels of CLDN3 and CLDN5, and a higher level of TJP1/ZO1in the ileum than Ctrl animals on day 44. In the caecum, weaning was associated with a lower expression of both CLDN3 and CLDN5 in Ctrl pigs, whereas lower expression levels were only observed for CLDN3 with overall lower level in ROM supplemented pigs. It has been reported that claudins can locate at TJs, as well as in the cytoplasm and on lateral membranes along the crypt-to-villus surface epithelial cells in the intestine [64], and alterations in the expression and localization of TJ proteins may cause intestinal disturbance [63]. Accumulating evidence suggests that increased expression of TJ protein encoding genes in the intestine is probably associated with improved intestinal integrity [65–69]. However, it should be noted that gene expression does not always reflect protein amounts and localisation in the TJ. Furthermore, a few studies have reported that expression of genes that code for TJ related proteins and/or mucins was also transiently increased in pigs challenged by heat stress or LPS [70–73]. Therefore, it is of interest for future studies to explore the regulatory mechanisms underlying the observed effects of ROM supplementation on the expression of genes associated with intestinal barrier function and to confirm observations at the protein level, including the distribution of the regulated claudins in intestinal epithelial cells. Additionally, the potential effect of short chain fatty acids (SCFAs) generated by gut microbiota on the TJ related proteins should not be excluded [74].

The epithelial TLR/IL-1R signalling pathway has been reported to play a crucial role in regulating gene expression and shaping host immune response in the intestine, and thus helping maintaining intestinal homeostasis [75, 76]. Weaning significantly accelerated expression of more genes related to TLR/IL-1R signalling in the ileal mucosa of ROM pigs, which may mount a more pronounced pro-inflammatory response in the ileum to weaning stress. In more detail, inclusion of ROM increased the expression of genes encoding two molecules TICAM2 and LY96 in the ileum on day 44, both of which are typically involved in TLR4 signalling [77]. LY96, also called myeloid differential protein-2 (MD-2), is an essential co-receptor and binds to the extracellular domain of TLR4, which is necessary for LPS to trigger TLR4 downstream signalling [78]. TICAM2, called TRIF-related adaptor molecule (TRAM), is involved in the TRIF–TRAM intracellular signalling pathway initiated by TLR4 [77]. This TRIF–TRAM signalling through TLR4 can result in a late phase NF-κB activation through activated interferon regulatory factor-3 (IRF3), as well as mediate the expression of type I interferon (IFN) and IFN-regulated genes [79, 80]. Furthermore, this TRIF–TRAM pathway through TLR4 has also been shown to influence dendritic cell (DC) function, and consequently influence the expression of co-stimulatory molecules, such as CD80 and CD86 [81]. Thus the effect of ROM supplementation on the ileal expression of genes CD80 CD86, NFKBIA, NFKB2, IL18, IL15, CXCL14, IL1A, CCL2 and CCL5 might be caused by TLR4 triggered TRIF–TRAM downstream signalling. In the caecum, ROM pigs had a higher expression of genes encoding the adaptor protein MYD88 and two negative regulatory proteins (TOLLIP and TNFAIP3) as compared to the ROM group on day 44. MYD88 is an essential adaptor protein for the TLR/IL-1R superfamily (except TLR3), and MYD88-dependent pathways can directly activate NF-κB and then drive the pro-inflammatory response via the production of pro-inflammatory cytokines, such as tumour necrosis factor (TNF) [79]. Furthermore, TOLLIP and TNFAIP3 are widely accepted as negative regulatory proteins involved in host immunological regulation by attenuating inflammatory responses triggered by TLRs [82, 83]. Those observations suggest that ROM supplementation early in life appears to lead to a heightened immune response in ileum with concomitant expression of negative regulators TOLLIP and TNFAIP3 to control inflammation during the critical post-weaning period.

Genes related to redox signalling and cell apoptosis were also modulated by ROM supplementation. Weaning stress has been reported to cause increased oxidative processes and then lead to enterocyte apoptosis, which negatively affects intestinal barrier function [1, 44]. In the current study, the expression of two genes involved in redox signalling, including GSTM3 and HIF1A, was significantly higher in the ileal mucosa only in ROM supplemented pigs on day 44 compared to day 27. However, the expression of these two genes was increased in the caecal mucosa in Ctrl pigs after weaning. Comparison between treatments further showed that inclusion of ROM resulted in an increased expression of HIF1A and a decreased expression of NOS2 in ileal mucosa, as well as an increased expression of PDGFRB in caecal mucosa on day 44 compared to Ctrl pigs. NOS2 and HIF1A have been widely accepted to be involved in maintenance of redox balance and linked to intestinal barrier function in the intestine mucosa [84, 85]. The expression of PDGFRB has also been reported to be associated with oxidative stress [86–88]. This could suggest that ROM supplementation early in life contributes to maintaining oxygen homeostasis in response to oxidative stress caused by weaning. The modulation of ROM supplementation on oxidative stress may be caused by a direct intestinal response to heightened immune responses or through intestinal microbiota and/or their metabolites (e.g., SCFAs) [89]. With respect to genes related to cell apoptosis, the expression level of pro-apoptotic BAX was significantly decreased in ROM treated pigs but increased in Ctrl animals on day 44 compared to day 27, whereas the expression level of an anti-apoptotic gene (BCL2) was increased in ROM pigs after weaning. Comparative analysis further revealed that the expression levels of BCL2 and BAX in the caecal mucosa of ROM pigs compared to Ctrl animals on day 44 were significantly increased and decreased, respectively. Such an increased BCL2/BAX ratio is considered the essential factor in the suppression of cell apoptosis [90–92]. This could further indicate the beneficial effect of ROM supplementation on preventing the intestinal epithelial cells from excessive apoptosis in response to weaning associated oxidative stress during the critical post-weaning transition period.

It is known that feed additives may influence the immune system either directly, or indirectly through the gut microbiota and epithelial barrier [93, 94]. In the current study, we observed a significant increase in IL-10 production by MLN cells and enhanced activation of NK cells during the pre-weaning period. However, no increase in vaccine-specific antibodies was observed. Though, a significant reduction of *Salmonella*-specific IgM was observed, which was already visible prior to vaccination (day 14). This may imply that *Salmonella*-specific antibodies were already present in the pigs’ blood or that antibodies to other *Enterobacteriaceae* cross-react with components of the ELISA. It has been suggested that the effects on the innate immune system are induced by pathogen-associated molecular patterns (PAMPs) such as β-glucans. PAMPs like β-glucans can interact with pattern recognition receptors (PRRs) on the surface of intestinal epithelial cells and innate immune cells (e.g., Dectin-1 and TLRs), thereby eliciting an immune response through specific signalling pathways [95, 96]. This is strengthened by our observation that genes involved in the Dectin-1/TLR signalling pathway (e.g., IRAK1, IRAK4, Myd88, NFKBIA, NFKB2, and TOLLIP) were significantly different between the treatment groups. In line with our findings, de Groot et al. demonstrated that ROM combined with mannan-rich hydrolysed copra meal increased the number of NK cells in porcine blood on day 15 post-weaning [11]. Another study that used gene-targeted mice and neutralizing monoclonal antibodies clearly showed that *A. subrufescens* augmented NK cell cytotoxicity through IL-12-mediated IFN-γ production [97]. Though, we did not observe a difference in IFN-γ gene expression between the treatment groups. The slight, but significant increase in IL-10 production by MLN cells upon ex vivo stimulation with LPS, also indicates that ROM influences cells of the innate immune system.

The observed effects of ROM supplementation early in life on piglet intestinal microbiota composition, epithelial gene expression, and host immune response could be linked to functional components either derived from *A. subrufescens* mycelia and/or rye during solid state fermentation. Recent studies have specified that antitumor, anti-inflammatory, antioxidant and antiallergic effects of the mycelium extract (Andosan™) mainly made from *A. subrufescens* are presumably due to β-glucans and low molecular weight (LMW) substances (e.g., isoflavonoids, blazein and ergosterol), although the quantity of the β-glucans is small and the main constituent of Andosan™ is still not fully resolved [7, 98]. A predominantly anti-inflammatory effect was observed from clinical studies, where volunteers [healthy controls or patients with ulcerative colitis (UC) or Crohn’s disease (CD)] who took Andosan™, showed a decrease in plasma levels of pro-inflammatory molecules (e.g., IL-1 β, IL-2, IL-5, IL-8, or calprotectin) [99–101]. An antioxidant action of extracts isolated from the mycelium of *A. subrufescens* was also observed in an in vitro assay [6], and in animal models [102, 103]. Additionally, extracts of *A. subrufescens* could also induce apoptosis as a mechanism to inhibit tumour cell growth both in vitro and in vivo [104, 105]. In the current study, ROM supplementation early in life affected gene expression with respect to effects on immunomodulation, redox signalling and apoptosis in intestinal epithelial cells. It could be speculated that these biological effects are, to a large extent, due to the compounds derived from *A. subrufescens* mycelium*.* However, some of the potentially beneficial effects from ROM could also be derived from rye compounds. Arabinoxylans as the main non-digestible fibre of cereal grains (rye included), were found to modulate gut microbiota and their metabolites (e.g., butyrate) as well as to enhance immune responses [106, 107].

## Conclusions

Taken together, the results observed in this study provide evidence that ROM supplementation for pigs in early life can positively modulate gut microbiota, intestinal mucosal function, and immune system development. This may contribute to improving health of pigs during the weaning period and further reducing antibiotics use. Further studies are required to clarify the constituents and functional compounds present in ROM, and to confirm if ROM supplementation in early life can indeed increase resilience of piglets against (intestinal) infections during the weaning period.

## Supplementary Information

**Additional file 1: Table S1.** Weaner and nursery diet. **Table S2.** Primers used for microfluidic qPCR. **Fig. S1.** The effect of ROM oral supplementation for pig on body weight in over time (**A**), average daily feed intake (**B**) and feed conversion ratio (**C**) in nursery phase. Control (Ctrl) and treatment (ROM) groups are represented by colour blue and yellow, respectively. Data is shown as the means ± the standard error of the mean (SEM). No significant differences on either body weight, average daily feed intake or feed conversion ratio were observed between both groups. **Fig. S2.** Comparison of faecal microbial alpha diversity between ROM and Ctrl groups over time, with metrics of observed richness (**A**), phylogenetic diversity (**B**), Shannon diversity (**C**) and inverse Simpson (InvSimpson) (**D**). Data was separated according to weaning for Linear Mixed-Effects Model analysis and no statistically significant differences were observed between the two treatment groups either during pre- or post-weaning phase, with any index of alpha diversity. Treatment (ROM) and control (Ctrl) groups are represented by colour yellow and blue, respectively. The vertical line indicates weaning on day 28. **Fig. S3.** Principal coordinate analysis (PCoA) of faecal microbial composition over time, based on unweighted UniFrac (**A**) and weighted UniFrac (**B**) distance metrics. Data was separated according to weaning on day 28 to assess the effect of time and treatment on microbial variation using PERMANOVA. Colours represent different time points, and shapes represent different treatment groups (circles, ROM; triangles, Ctrl). The percentages at the axes indicate the variation explained. **Fig. S4.** Genera showing tendencies to be differential abundant between ROM and Ctrl pigs during pre-or post weaning. Genera were identified through GAMLSS-BEZI model with random effect. The *p* value was corrected by FDR for multiple testing. The yellow and blue colours represent treated (ROM) and control (Ctrl) groups, respectively. **Fig. S5.** Comparisons of gut luminal microbial alpha diversity between ROM) and Ctrl groups over time. The jejunal, ileal and caecal luminal microbial alpha diversity are shown in (**A**–**D**), (**E**–**H**) and (**I**–**L**), respectively, with metrics of observed richness, phylogenetic diversity, Shannon diversity and inverse Simpson (InvSimpson), from left to right. Differences were assessed with a Linear Mixed-Effects Model for jejunal, ileal and caecal luminal microbial alpha diversity for all time points, respectively. Treatment (ROM) and control (Ctrl) groups are represented by colour yellow and blue, respectively. **Fig. S6.** Principal coordinate analysis (PCoA) plots for the overall microbial composition of intestinal luminal digesta at different locations, based on unweighted- and weighted UniFrac metrics. Significance of the effect of time and treatment on jejunal- (**A**, **B**), ileal- (**C**, **D**) and caecal (**E**, **F**) luminal microbiota at ASV level was assessed by PERMANOVA. Colours represent different time points, and shapes represent different treatment groups (circles, ROM; triangles, Ctrl). The percentages at the axes indicate the variation explained. **Fig. S7.** Bar plots representing relative abundance in individual pig jejunal (**A**–**C**) and caecal (**D**–**F**) luminal contents of specific taxa identified as differential features by LEfSe for pigs between control (class: Ctrl) and treatment (class: ROM) on day 70. The solid black horizontal line indicates the mean relative abundance within each class. **Fig. S8.** Redundancy analysis (RDA) tri-plots for the association between the variation of gene expression and environmental variables of time, treatment, pH and BW. **A** and **B** illustrates the association between ileal and caecal mucosa expressed genes with environmental variables of time, treatment, pH value and body weight (BW), respectively. **C** shows the association between the caecal mucosal gene expression and treatment, pH values and BW. Blue and yellow colours represent control (Ctrl) and treated (ROM) groups and shapes show the different time points. Dark grey arrows indicate environmental variables and light grey arrows show best fitting genes in the model. The percentages at the axes indicate the variation explained by the first two canonical axes. **Fig. S9.** Correlation analysis between the relative abundance of predominant ileal luminal genera and mucosal gene expression on day 27. The orange and blue coloured circles correspond to a positive and negative statistical correlation with an adjusted *p* value < 0.05, respectively. Insignificant correlations were shown as blank. **Fig. S10.** Correlation analysis between the relative abundance of predominant ileal luminal genera and mucosal gene expression on day 44. The orange and blue coloured circles correspond to a positive and negative statistical correlation with an adjusted *p* value < 0.05, respectively. **Fig. S11.** Correlation analysis between the relative abundance of predominant caecal luminal genera and mucosal gene expression on day 27. The orange and blue coloured circles correspond to a positive and negative statistical correlation with an adjusted *p* value < 0.05, respectively. Insignificant correlations were shown as blank. **Fig. S12.** Correlation analysis between the relative abundance of predominant caecal luminal genera and mucosal gene expressions on day 44. The orange and blue coloured circles correspond to a positive and negative statistical correlation with an adjusted *p* value < 0.05, respectively. Insignificant correlations were shown as blank.

## Data Availability

The sequence data was submitted to European Nucleotide Archive with accession number PRJEB54013.

## Abbreviations

NK cells: Natural killer cells
TLR4: Toll-like receptor 4
CD: Cluster of differentiation
CLDN3: Claudin 3
MUC2: Mucin 2
MYD88: Myeloid differentiation primary response protein 88
NOS2: Nitric oxide synthase 2
HIF1A: Hypoxia-inducible factor 1-alpha
TOLLIP: Toll interacting protein
BCL2: B-cell lymphoma 2
BAX: BCL2 Associated X, apoptosis regulator
TICAM2: Toll like receptor adaptor molecule 2
LY96: Lymphocyte antigen 96 (MD2)
NFKBIA: Nuclear factor of kappa light polypeptide gene enhancer in B-cells inhibitor, alpha
TNF: Tumor necrosis factor alpha
GLUT3: Solute carrier family 2 member 3
NFKB1: Nuclear factor kappa b subunit 1
IRAK4: Interleukin-1 receptor-associated kinase 4
TNFAIP3: Tumor necrosis factor, alpha-induced protein 3
IKBKB: Inhibitor of nuclear factor kappa-B kinase subunit beta, IKK-β
PERMANOVA: Permutational multivariate analysis of variance
