# Supplementary material for: Agaricus subrufescens fermented rye affects the development of intestinal microbiota, local intestinal and innate immunity in suckling-to-nursery pigs
Source: Anim Microbiome. 2023 Apr 11;5:24. doi: 10.1186/s42523-023-00244-w (PMC10088699; doi:10.1186/s42523-023-00244-w)
Supplement: Supplementary file 1 — Additional file 1: Table S1. Weaner and nursery diet. Table S2. Primers used for microfluidic qPCR. Fig. S1. The effect of ROM oral supplementation for pig on body weight in over time (A), average daily feed intake (B) and feed conversion ratio (C) in nursery phase. Control (Ctrl) and treatment (ROM) groups are represented by colour blue and yellow, respectively. Data is shown as the means ± the standard error of the mean (SEM). No significant differences on either body weight, average daily feed intake or feed conversion ratio were observed between both groups. Fig. S2. Comparison of faecal microbial alpha diversity between ROM and Ctrl groups over time, with metrics of observed richness (A), phylogenetic diversity (B), Shannon diversity (C) and inverse Simpson (InvSimpson) (D). Data was separated according to weaning for Linear Mixed-Effects Model analysis and no statistically significant differences were observed between the two treatment groups either during pre- or post-weaning phase, with any index of alpha diversity. Treatment (ROM) and control (Ctrl) groups are represented by colour yellow and blue, respectively. The vertical line indicates weaning on day 28. Fig. S3. Principal coordinate analysis (PCoA) of faecal microbial composition over time, based on unweighted UniFrac (A) and weighted UniFrac (B) distance metrics. Data was separated according to weaning on day 28 to assess the effect of time and treatment on microbial variation using PERMANOVA. Colours represent different time points, and shapes represent different treatment groups (circles, ROM; triangles, Ctrl). The percentages at the axes indicate the variation explained. Fig. S4. Genera showing tendencies to be differential abundant between ROM and Ctrl pigs during pre-or post weaning. Genera were identified through GAMLSS-BEZI model with random effect. The p value was corrected by FDR for multiple testing. The yellow and blue colours represent treated (ROM) and control (Ctrl) groups, respectively. [file 42523_2023_244_MOESM1_ESM.docx]

**Tab. S1 Weaner and nursery diet**

|  | **Weaner Diet**  Day 25-44 | **Nursery Diet**  Day 45-70 |
| --- | --- | --- |
| *Ingredient composition (%)* |  |  |
| Barley | 25.00 | 30.00 |
| Wheat | 25.00 | 22.00 |
| Corn | 15.28 | 12.00 |
| Soybean meal (48% crude protein) | 10.00 | 9.90 |
| Sweet whey powder | 9.29 | 4.29 |
| Soy protein concentrate ^1^ | 4.30 | 4.00 |
| Soya oil | 3.20 | 3.31 |
| Cane molasses (> 47.5% sugar) | 1.00 | 1.00 |
| Sucrose | 1.50 | 1.50 |
| Wheat bran | 1.50 | 5.13 |
| Sunflower seed meal (27% crude protein) | 0 | 3.00 |
| Sodium chloride | 0.58 | 0.46 |
| Sodium bicarbonate | 0 | 0.29 |
| Mono-calcium phosphate | 0.29 | 0.03 |
| Limestone (calcium carbonate) | 0.47 | 0.56 |
| Organic acids ^2^ | 0.15 | 0.15 |
| Phytase ^3^ | 0.05 | 0.05 |
| Vitamins and trace minerals ^4^ | 1.05 | 1.12 |
| Synthetic amino acids | 1.34 | 1.21 |
| Total | 100.00 | 100.00 |
|  |  |  |
| *Calculated nutrients, g/kg* |  |  |
| Moisture | 110 | 113 |
| Crude protein | 170 | 175 |
| Crude fat | 50 | 52 |
| Crude fibre | 28 | 40 |
| Crude ash | 47 | 47 |
| Starch (Ewers method) | 384 | 378 |
| Total dietary fibre | 141 | 165 |
| Soluble dietary fibre | 64 | 79 |
| Insoluble dietary fibre | 80 | 94 |
| Lactose | 65 | 30 |
| Calcium | 5.50 | 5.50 |
| Phosphorus | 4.50 | 4.36 |
| Digestible Phosphorus | 4.10 | 3.50 |
| Na | 3.00 | 3.00 |
| Cu (total, mg) | 165 | 167 |
| Zn (total, mg) | 124 | 129 |
| Metabolic energy (MJ) | 14.22 | 13.84 |
| Net energy (MJ) | 10.46 | 10.11 |
| Standardized ileal digestible lysine | 12.35 | 12.03 |
| *Analysed nutrients, g/kg* |  |  |
| Moisture | 101 | 97 |
| Crude protein | 170 | 176 |
| Crude fibre | 28 | 40 |
| Crude fat | 53 | 53 |
| Crude ash | 46 | 47 |
| Zinc (mg/kg) | 118 | 116 |

^1^HP 300 (Hamlet protein, Horsens, Denmark); ^2^ Fylax Forte HC-SP (Trouw Nutrition Selko, Tilburg, The Netherlands) ^3^ Phyzyme XP 5000 TPT (Danisco Animal Nutrition, Marlbourough, UK ) providing 600 FTU 6-phytase per kg feed; ^4^ Farmix (Trouw Nutrition, Putten, The Netherlands), provided per kg feed: 8000 IU vit A, 2000 IU vit D3, 100 (weaner) or 150 (nursery) IU vit E-acetate, 1.5 mg menadione, 1 mg thiamine mononitrate, 4 mg riboflavin, 1 mg pyridoxine, 30 µg cyanocobalamin, 20 mg niacin, 12 mg pantothenic acid, 300 µg folic acid, 150 mg choline chloride, 50 mg betain.

**Tab. S2 Primers used for microfluidic qPCR**

| **Primers** | **Gene name** | **Forward primer sequence (5' to 3')** | **Reverse primer sequence (5' to 3')** | **Amplicon length** | **Primer efficiency** |
| --- | --- | --- | --- | --- | --- |
| ACTB | Actin beta | CTACGTCGCCCTGGACTTC | GCAGCTCGTAGCTCTTCTCC | 76 | 1.13 |
| B2M | Beta-2-microglobulin | TGAAGCACGTGACTCTCGAT | CTCTGTGATGCCGGTTAGTG | 70 | 1.13 |
| C3 | Complement Component 3 | ATCAAATCAGGCTCCGATGA | GGGCTTCTCTGCATTTGATG | 76 | 1.13 |
| C5 | Complement Component 5 | AAGCTGGAGAAGCCGTTGC | TTTTCGAGGTTAGCGTTCGT | 82 | 1.15 |
| DEFB1 (15) | Defensin Beta 1 | ACCTGTGCCAGGTCTACTAAAAA | GGTGCCGATCTGTTTCATCT | 109 | 1.13 |
| GAPDH | Glyceraldehyde-3-phosphate dehydrogenase | ACCCAGAAGACTGTGGATGG | AAGCAGGGATGATGTTCTGG | 79 | 1.13 |
| HPRT1 | Hypoxanthine phosphoribosyltransferase 1 | ACACTGGCAAAACAATGCAA | TGCAACCTTGACCATCTTTG | 71 | 1.13 |
| IL1B | Interleukin 1b | CCAAAGAGGGACATGGAGAA | GGGCTTTTGTTCTGCTTGAG | 123 | 1.14 |
| IL6 | Interleukin 6 | TGGGTTCAATCAGGAGACCT | CAGCCTCGACATTTCCCTTA | 116 | 1.08 |
| IL8 | Interleukin 8 | GAAGAGAACTGAGAAGCAACAACA | TTGTGTTGGCATCTTTACTGAGA | 99 | 1.18 |
| IL12A | Interleukin 12 p35 | GAACTAGCCACGAATGAGAGTTG | ACTGCTAAGGCACAGGGTTG | 114 | 1.13 |
| IL12B | Interleukin 12 p40 | GACCAGAAAGAGCCCAAAAAC | AGGTGAAACGTCCGGAGTAA | 70 | 1.04 |
| IFNA | Interferon alpha 1 | ATCGTCAGGGCAGAAGTCAT | CCAGGTGTCTGTCACTCCTTC | 86 | 1.17 |
| IFNG | Interferon gamma | CCATTCAAAGGAGCATGGAT | TTCAGTTTCCCAGAGCTACCA | 76 | 1.14 |
| RPL13A | Ribosomal protein L13a | ATTGTGGCCAAGCAGGTACT | AATTGCCAGAAATGTTGATGC | 76 | 1.13 |
| SAA2 | Serum amyloid A2 |    \| TAAAGTGATCAGCAATGCCAAA \| \| --- \| | TCAACCCTTGAGTCCTCCAC | 96 | 1.13 |
| TGFB1 | Transforming Growth Factor Beta 1 | TCACCGGGGCTGTATTTAAG | AAGGAAGACCCCAGTCAGGT | 110 | 1.13 |
| TNF | Tumor Necrosis Factor alpha | CCCCCAGAAGGAAGAGTTTC | CGGGCTTATCTGAGGTTTGA | 92 | 1.16 |
| LBP | Lipopolysaccharide Binding Protein | CCCAAGGTCAATGATAAGTTGG | ATCTGGAGAACAGGGTCGTG | 83 | 1.11 |
| TLR9 | Toll like receptor 9 | CCTGTTCTATGATGCCTTCGTG | GGTACCCAGTCTCGCTCCTC | 144 | 1.13 |
| TLR2 (100) | Toll like receptor 2 | GTTTTACGGAAATTGTGAAACTG | TCCACATTACCGAGGGATTT | 136 | 1.14 |
| IL13 | Interleukin 13 | CCAAGCGAGCAAGTTCCTG | AACTACCCGTGGCGAAAAAT | 110 | 1.10 |
| IL27 | Interleukin 27 | GCCACTTTGCTGAATCACAC | TGGAGAGGAAGCAGAGTCGT | 135 | 1.13 |
| CXCL10 | Chemokine (C-X-C Motif) Ligand 10 | CCCACATGTTGAGATCATTGC | GCTTCTCTCTGTGTTCGAGGA | 141 | 1.13 |
| CCL5 | C-C Motif Chemokine Ligand 5 | CTCCATGGCAGCAGTCGT | AAGGCTTCCTCCATCCTAGC | 121 | 1.13 |
| TLR3 (123) | Toll like receptor 3 | ATTGTGCAAAAGATTCAAGGTG | TCTTCGCAAACAGAGTGCAT | 130 | 1.17 |
| TLR7 | Toll like receptor 7 | AGAAGCCCCTTCAGAAGTCC | GGTGAGCCTGTGGATTTGTT | 93 | 1.13 |
| NFKBIA | Nuclear Factor Of Kappa Light Polypeptide Gene Enhancer In B-Cells Inhibitor, Alpha |    \| GAGGATGAGCTGCCCTATGAC \| \| --- \| | CCATGGTCTTTTAGACACTTTCC | 85 | 1.18 |
| IL10 | Interleukin 10 | TACAACAGGGGCTTGCTCTT | GCCAGGAAGATCAGGCAATA | 110 | 1.13 |
| MUC2 | Mucin 2 | GCACGTCTGCAACAAGGAC | CAAAGCCCTCCAGGCAGT | 125 | 1.13 |
| CLDN3 | Claudin 3 | ATCGGCAGCAGCATTATCAC | ACACTTTGCACTGCATCTGG | 94 | 1.13 |
| IL1RN | Interleukin 1 Receptor Antagonist | TGCCTGTCCTGTGTCAAGTC | GTCCTGCTCGCTGTTCTTTC | 90 | 1.13 |
| TNFAIP3 | Tumor Necrosis Factor, Alpha-Induced Protein 3 | CCCAGCTTTCTCTCATGGAC | TTGGTTCTTCTGCCGTCTCT | 113 | 1.13 |
| PPIA | Peptidylprolyl isomerase A (cyclophilin A) | CAAGACTGAGTGGTTGGATGG | TGTCCACAGTCAGCAATGGT | 138 | 1.13 |
| YWHAE | Tyrosine 3-monooxygenase/tryptophan 5-monooxygenase | GCTGCTGGTGATGATAAGAAGG | AGTTAAGGGCCAGACCCAAT | 124 | 1.13 |
| IL1A |    \| Interleukin 1 alpha \| \| --- \| | TGTGCTAAATAACCTGGATGAGG | GGTTCGTCTTCGTTTTGAGC | 135 | 1.16 |
| SAA |    \| Serum Amyloid A \| \| --- \| | GCTAAAGTGATCAGCGATGC | AGTGGTTGGGGTCCTTGC | 145 | 1.13 |
| TLR2 (160) | Toll like receptor 2 | CGGAGGTTGCATATTCCACAG | TGTGAAAGGGAACAGGGAAC | 128 | 1.18 |
| TLR3 (161) | Toll like receptor 3 | ACATCTACTGAAAGATCCATTGTGC | TCTTCGCAAACAGAGTGCAT | 148 | 1.12 |
| TLR6 | Toll like receptor 6 | TGGATGTTAGCTCGAATTCTTTG | GAACCTTGATCCTGGGAGGT | 141 | 1.13 |
| LY96 | Lymphocyte Antigen 96 (MD2) | CAGTAAAGGTTGAGCCCTGTG | TTTGCGCATTGGTAAAGTCA | 140 | 1.19 |
| MYD88 | Myeloid differentiation primary response protein 88 | CCAGACTAAGTTTGCACTCAGC | AGGATGCTGGGGAACTCTTT | 99 | 1.11 |
| TLR8 | Toll like receptor 8 | GCAAAGACCACCACCAACTT | ATCCGTCAGTCTGGGAATTG | 129 | 1.07 |
| TLR5 | Toll like receptor 5 | AGTTCTGAACCTGGCCTTCA | TAAGCGAGCTTAGGCAGTCC | 144 | 1.13 |
| TLR1 | Toll like receptor 1 | CCTTCAAGACCTTAACACACAGAG | CAGATTTACTGCGGTGCTGA | 100 | 1.17 |
| IL23A | Interleukin 23 | GCTGTGATCCTCAGGGACTC | TAGAGAAGGCTCCCCTGTGA | 119 | 1.13 |
| MUC3A | Mucin 3A, cell surface associated | AACTTGTTCCGCTTCTCGAC | CCAGGCTCGACTCTTAGGTG | 129 | 1.13 |
| TFF2 | Trefoil Factor 2 | GCTGCTTCGACTCCCAAGT | CATGACGCACTCCTCAGACT | 80 | 1.13 |
| TFF3 | Trefoil Factor 3 | TGTTCTGGCTGCTAGTGGTG | CAGTCCACCCTGTCCTTGG | 112 | 1.13 |
| IL18 | Interleukin 18 | CAATTGCATCAGCTTTGTGG | TCCAGGTCCTCATCGTTTTC | 78 | 1.14 |
| TLR4 | Toll like receptor 4 | TGGTGTCCCAGCACTTCATA | CAACTTCTGCAGGACGATGA | 116 | 1.18 |
| SAA 2 AND 3 | Serum Amyloid A | CAGAGATGGGCATCATTCCT | TGGCATCGCTGATCACTTTA | 184 | 1.13 |
| NOS2 | Nitric Oxide Synthase 2 | GCAGCTACTGGGTCAAGGAC | GCTGTTGGTGAACTTCCACTT | 200 | 1.13 |
| CXCL14 | C-X-C Motif Chemokine Ligand 14 | GTACCGAGGTCAGGAGCACT | TAGACCCTGCGCTTCTCATT | 96 | 1.13 |
| IL15 | Interleukin 15 | CGTCATTTTGCAAGAGTCCA | TGGACGATAAACTGCTGTTTGC | 86 | 1.16 |
| CCL2 | C-C Motif Chemokine Ligand 2 | CTTCTGCACCCAGGTCCTT | CGCTGCATCGAGATCTTCTT | 93 | 1.15 |
| IDO1 | Indoleamine 2,3-dioxygenase 1 | GGGCCCATGACTTACAAGAA | TTTCCACCAATAGCGAAACC | 94 | 1.12 |
| IL10RB | interferon alpha and beta receptor subunit 2 (IFNAR2) | TTCAAGTCCGAGCGTTTCTT | GGTTTCGTCATTGGTCGTCT | 86 | 1.13 |
| OCLN | Occludin | GACGAGCTGGAGGAAGACTG | GTACTCCTGCAGGCCACTGT | 102 | 1.15 |
| CLDN1 | Claudin 1 | GGTCAGGCTCTCTTCACTGG | ATGTTGTTTTTCGGGGACAG | 99 | 1.13 |
| NOD1 | Nucleotide-binding oligomerization domain-containing protein 1 | CAGTGGGGTGAAGGTGCTAT | TACCTGGCTCCGACATCAGT | 99 | 1.13 |
| CASP3 | Caspase 3 | CTGGCAAACCCAAACTTTTC | GTCCCACTGTCCGTCTCAAT | 79 | 1.15 |
| TJP1/ZO1 | Tight Junction Protein 1 | ATGACTCCTGACGGTTGGTC | TGCCAGGTTTTAGGATCACC | 71 | 1.10 |
| GLUT3/SLC2A3 | Solute Carrier Family 2 Member 3 | TCCCCTCAGCTGCATTCTAT | CCAGAAGACAACGAGGAAGC | 71 | 1.14 |
| CLDN5 | Claudin-5 | CTGGTTCGCCAACATCGT | AAGCTTCTCCTGCTCTGCTG | 70 | 1.19 |
| CALB1 | Calbindin 1 | GGGCAAAGAGATGATGGAAA | ATCGGAATAGCAGCAGGAAA | 85 | 0.88 |
| PDGFRB | Platelet-derived growth factor receptor beta | CTCACCGTCATCTCCCTCAT | AGCTCACGGATTCGATCACT | 91 | 1.19 |
| TP53 (493) | Tumor protein p53 | AAATCCGCTTCACCATCAAG | CAGGCTCCCATTGGATGTAG | 107 | 1.13 |
| TP53 (494) | Tumor protein p53 | TGACCATCTTTTCCCTCCTG | GGGCAGATGATGTCCAGTTT | 91 | 1.13 |
| IRAK1 | Interleukin-1 receptor-associated kinase 1 | GGATGGGGTTCTGGACAGC | TTCATCACTCTCTTCGGGCC | 100 | 1.13 |
| FAS | Fas receptor | GGTGAAAAGACGGTGCAGAAG | ACGTCTTTTATCATTGGCACCTC | 87 | 1.19 |
| FASLG | Fas ligand | CACCCCAATCTACCCTCTGAG | GTGTCTTCCCATTCCAGAGGG | 98 | 1.02 |
| NFKB2 (538) | Nuclear Factor Kappa B Subunit 2 | CTGAGACACGAGAGAAGCTGC | CTCTGCTTCCTGTTCCACCG | 86 | 1.13 |
| NFKB2 (539) | Nuclear Factor Kappa B Subunit 2 | CCAAAAGGATGAGCTGAAGTCC | CAGCCCCAATTGTCTCACCT | 115 | 1.06 |
| TOLLIP | Toll Interacting Protein | GACCCGTACTGCCGACTG | ATCACCTTATTCCAGCGCGG | 92 | 1.18 |
| IKBKB | Inhibitor of nuclear factor kappa-B kinase subunit beta, IKK-β | AAGAGCAGGCACGGGAAC | CCATTTCCTGACTGTCGCCA | 81 | 1.13 |
| TICAM2 | toll like receptor adaptor molecule 2 | TCTGCTGCAAAATGACTTCGG | AGCCATTGACAGCATCGTCT | 101 | 1.14 |
| CD86 | Cluster of differentiation 86 | CATCGTCTGTGTCCTGCAAC | CACAGGTGGCTTTGCATCTA | 82 | 1.16 |
| ID2 | Inhibitor Of DNA Binding 2, Dominant Negative Helix-Loop-Helix Protein | CCAGTGAGGTCCGTTAGGAA | GTTGTACAGCAGGCTCATCG | 99 | 1.13 |
| NFKB1 | Nuclear Factor Kappa B Subunit 1 | CCCTGTGAAGACCACCTCTC | ATCCCGGAGCTCGTCTATTT | 82 | 1.13 |
| BCL2 | Bcl-2 (B-cell lymphoma 2) | CCCTGTGGATGACTGAGTACC | AACCACACATGCACCTACCC | 83 | 1.18 |
| TICAM1 | Toll like receptor adaptor molecule 1 | CTGCCTTCCCACAGCCTC | AGCCCCAGTTGTACCATTTGA | 109 | 1.13 |
| GZMA | Granzyme A | AAGGGGATCTTCAGCTGCTT | GGGGTTCGACATCTTTTCCT | 99 | 1.17 |
| SLC2A5 | Solute Carrier Family 2 Member 5 | GGTCATCTCCACCATCATCC | GCGCTCAGGTAGATCTGGTC | 90 | 1.20 |
| DEFB1(179) | Beta-defensin 1 | TTCCTCCTCATGGTCCTGTT | CATCTTTGGAGCACACTTGC | 114 | 1.05 |
| RETNLB | Resistin Like Beta | TCCCTCTGCTCCAAGAAAGA | CAAGCACAGCCAGTGACAAC | 99 | 1.13 |
| GSTM3 | Glutathione S-Transferase Mu 3 | TCCTGGGGAAATACTCATGG | TGGGCTCAAAGATACGGTTC | 96 | 1.14 |
| CD80 (1063) | Cluster of differentiation 80 | CGCACCTTCACTGATGTCAC | CACAGGTGTAGGTGCCATTG | 82 | 1.21 |
| CD80 (1064) | Cluster of differentiation 80 | TTTCAATGTGACAGGCAACC | TGATTAGCAGAAGAGGTTTCTCG | 177 | 1.13 |
| GATA3 | GATA Binding Protein 3 | CTGGAGAAGAAATGCCAACG | CTTCATGGTCAGGGGTCTGT | 91 | 0.99 |
| HIF1A | Hypoxia-inducible factor 1-alpha | TTACAGCAGCCAGATGATCG | TCTTTTGCTCCGTTCCATTC | 97 | 1.13 |
| IL1R1 | Interleukin -1 receptor like 1 | CTCCCGGGTGATAAAACTGA | CCCAAGATCACTTCCATCGT | 96 | 1.10 |
| IRAK4 | Interleukin-1 receptor-associated kinase 4 | CGACTGTCTTGCTTGGATGA | ACTGAGGCCATTAGCTGCAC | 84 | 1.15 |
| BAX | BCL2 Associated X, Apoptosis Regulator | TGCCAGTAAACTGGTGCTCA | CGATCTCGAAGGAAGTCCAG | 93 | 1.12 |
| SLC5A8 | Solute carrier family 5 member 8 | TGGGACAAATTGGATGACAA | CCATCAGTGGAGTCCTTTCAA | 86 | 1.07 |
| MUC1 | Mucin 1 | GGCAGTACCAAACGGAACC | GCTGCCAGGTTCGAGTAAGA | 80 | 1.13 |


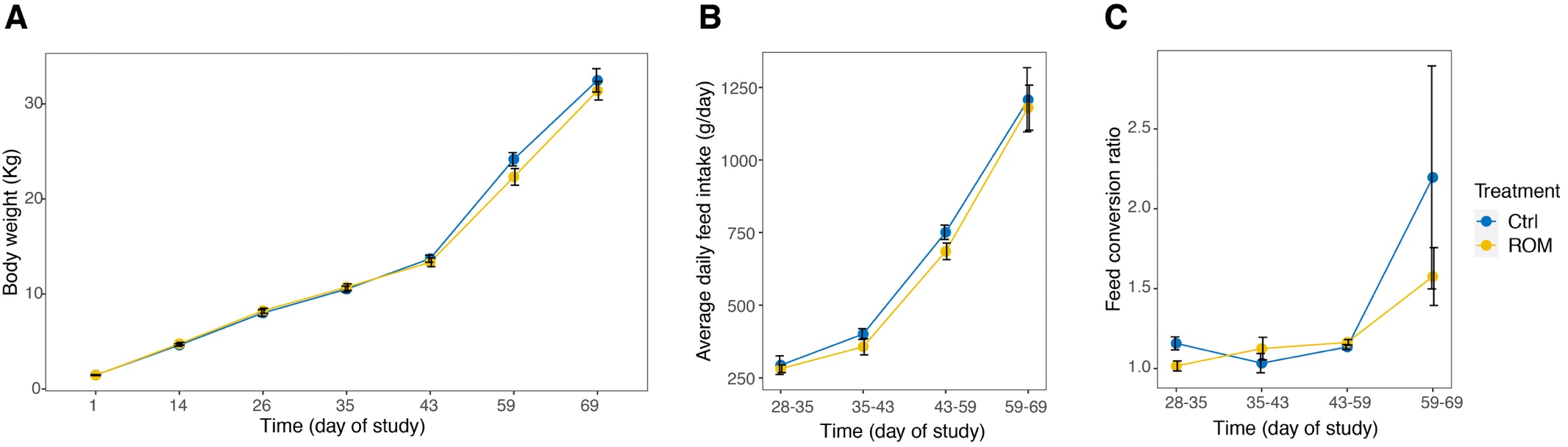


**Fig. S1 The effect of ROM oral supplementation for pig on body weight in over time (A), average daily feed intake (B) and feed conversion ratio (C) in nursery phase.** Control (Ctrl) and treatment (ROM) groups are represented by colour blue and yellow, respectively. Data is shown as the means ± the standard error of the mean (SEM). No significant differences on either body weight, average daily feed intake or feed conversion ratio were observed between both groups


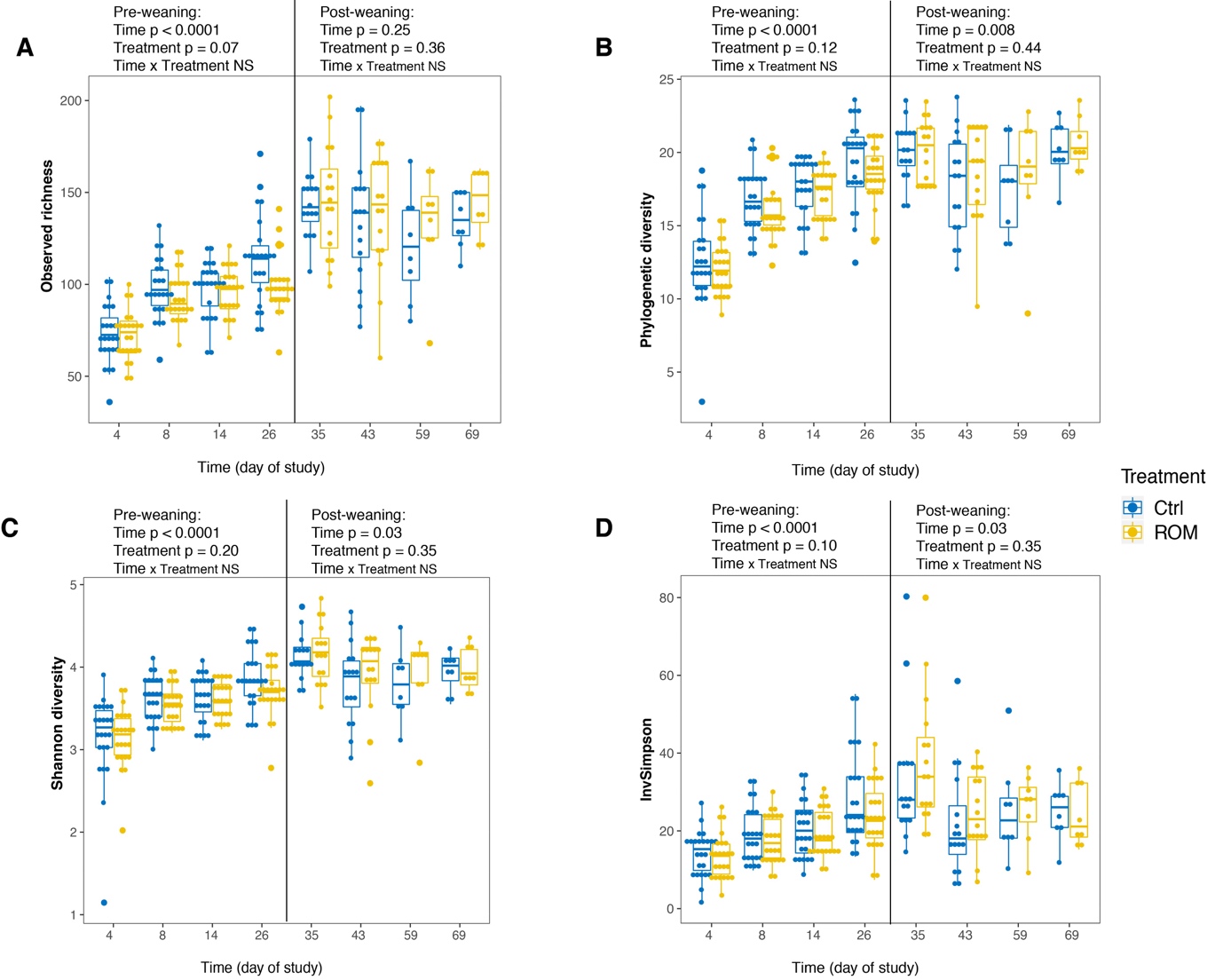


**Fig. S2 Comparison of faecal microbial alpha diversity between ROM and Ctrl groups over time, with metrics of observed richness (A), phylogenetic diversity (B), Shannon diversity (C) and inverse Simpson (InvSimpson) (D).** Data was separated according to weaning for Linear Mixed-Effects Model analysis and no statistically significant differences were observed between the two treatment groups either during pre- or post-weaning phase, with any index of alpha diversity. Treatment (ROM) and control (Ctrl) groups are represented by colour yellow and blue, respectively. The vertical line indicates weaning on day 28.


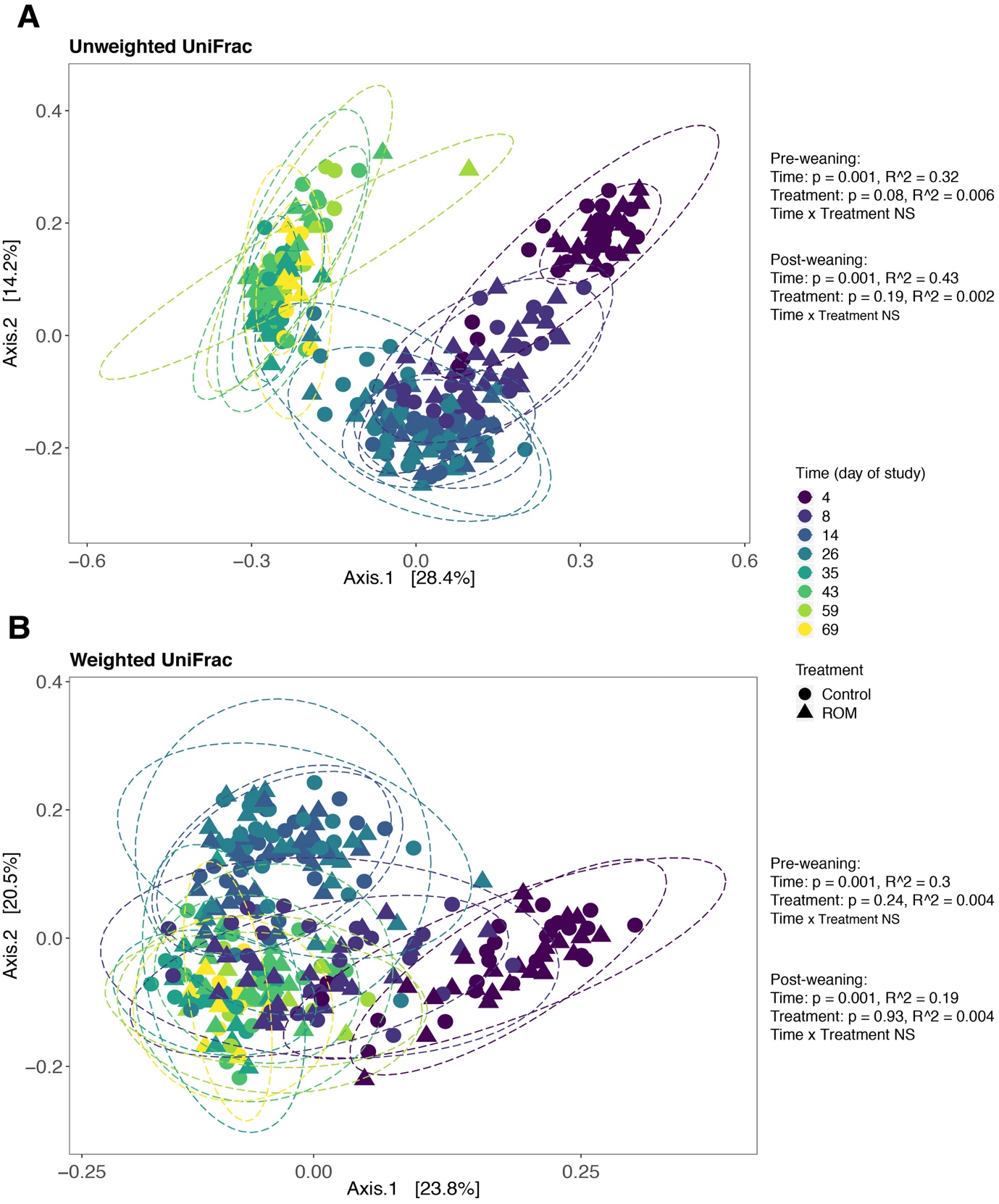


**Fig. S3 Principal coordinate analysis (PCoA) of faecal microbial composition over time, based on unweighted UniFrac (A) and weighted UniFrac (B) distance metrics.** Data was separated according to weaning on day 28 to assess the effect of time and treatment on microbial variation using PERMANOVA. Colours represent different time points, and shapes represent different treatment groups (circles, ROM; triangles, Ctrl). The percentages at the axes indicate the variation explained.


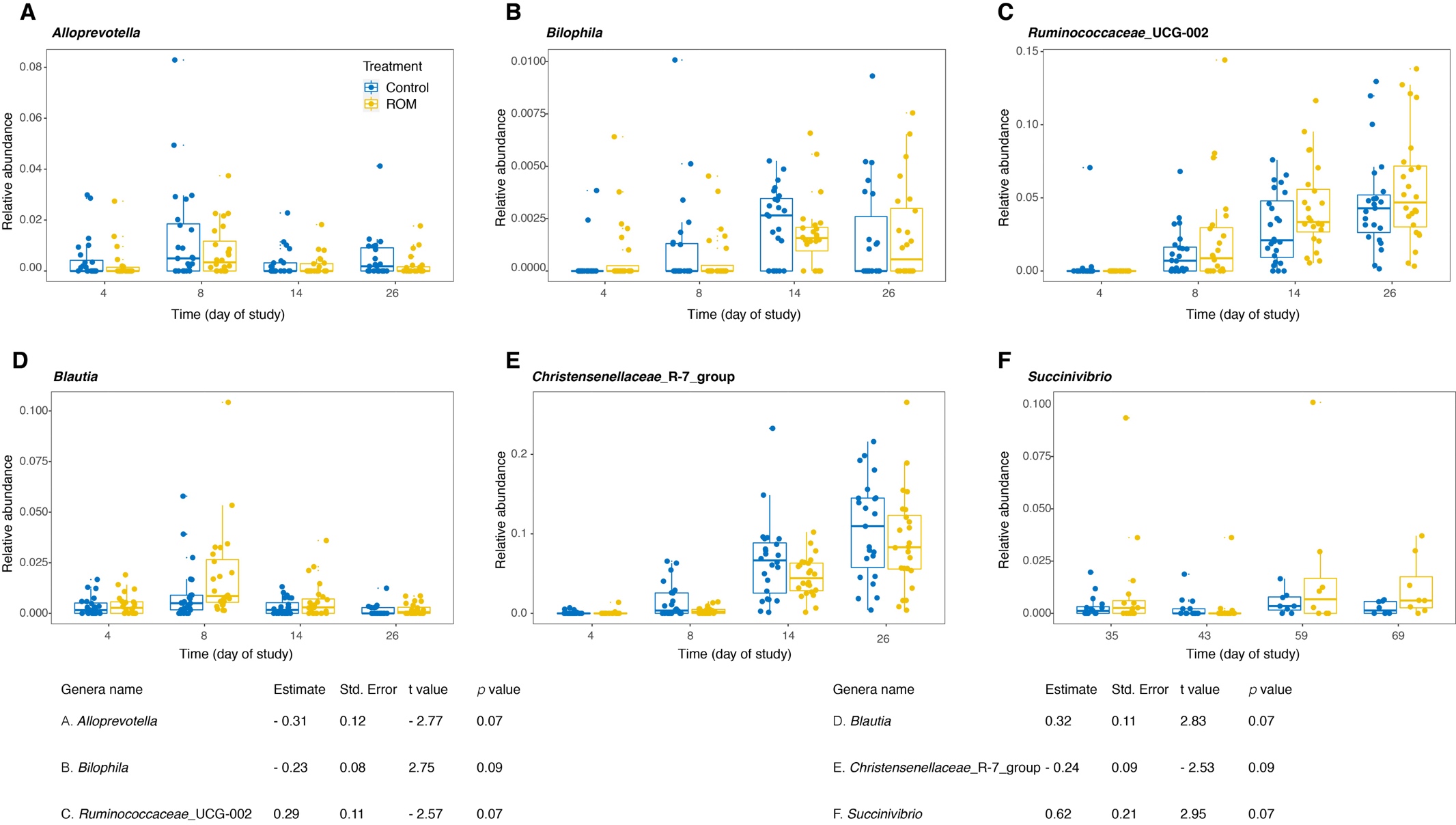


**Fig. S4** **Genera showing tendencies to be differential abundant between ROM and Ctrl pigs during pre-or post weaning.** Genera were identified through GAMLSS -BEZI model with random effect. The *p* value was corrected by FDR for multiple testing. The yellow and blue colours represent treated (ROM) and control (Ctrl) groups, respectively.


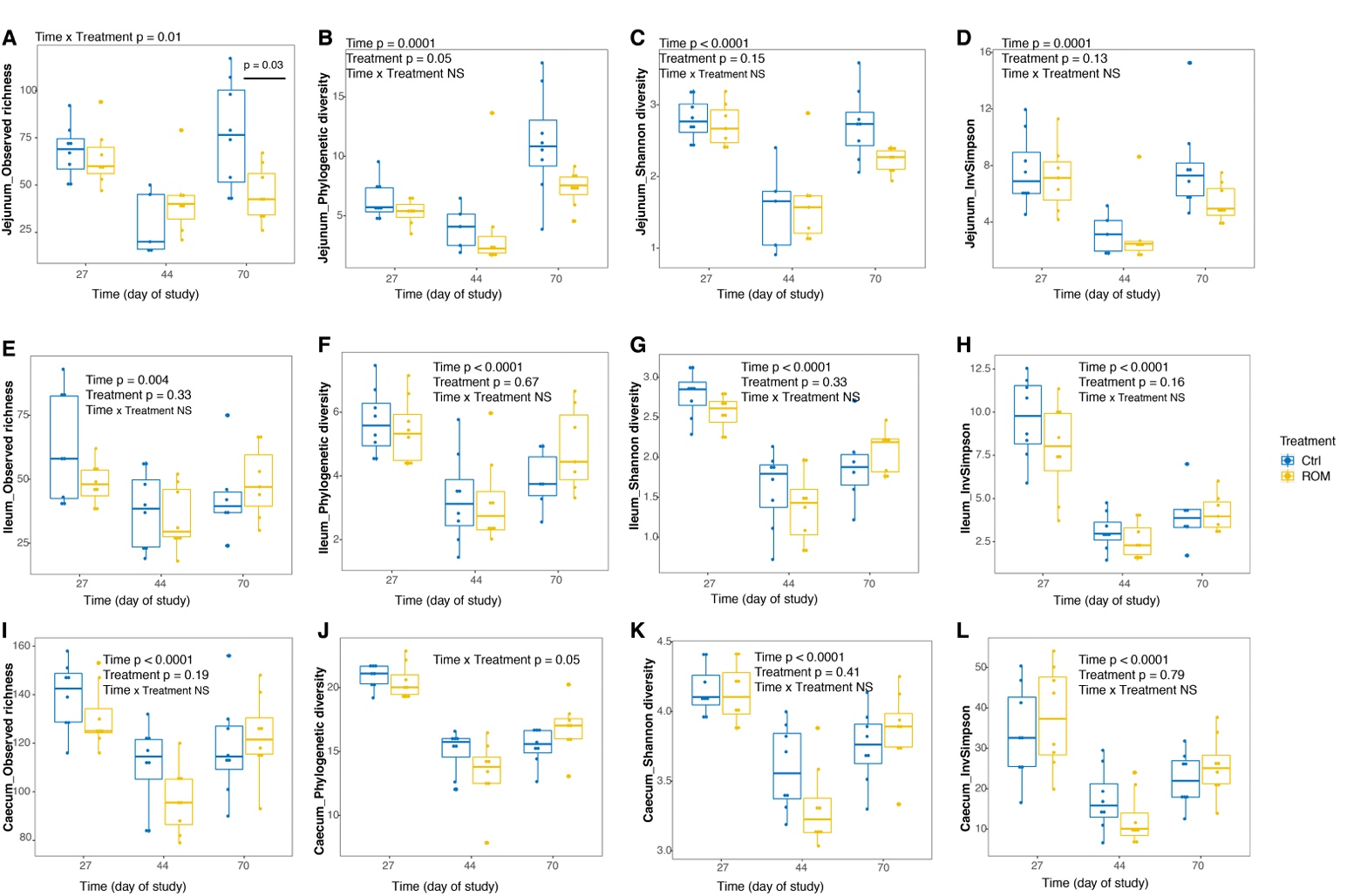


**Fig. S5 Comparisons of gut luminal microbial alpha diversity between ROM) and Ctrl groups over time.** The jejunal, ileal and caecal luminal microbial alpha diversity are shown in (**A-D**), (**E-H**) and (**I-L**), respectively, with metrics of observed richness, phylogenetic diversity, Shannon diversity and inverse Simpson (InvSimpson), from left to right. Differences were assessed with a Linear Mixed-Effects Model for jejunal, ileal and caecal luminal microbial alpha diversity for all time points, respectively. Treatment (ROM) and control (Ctrl) groups are represented by colour yellow and blue, respectively.


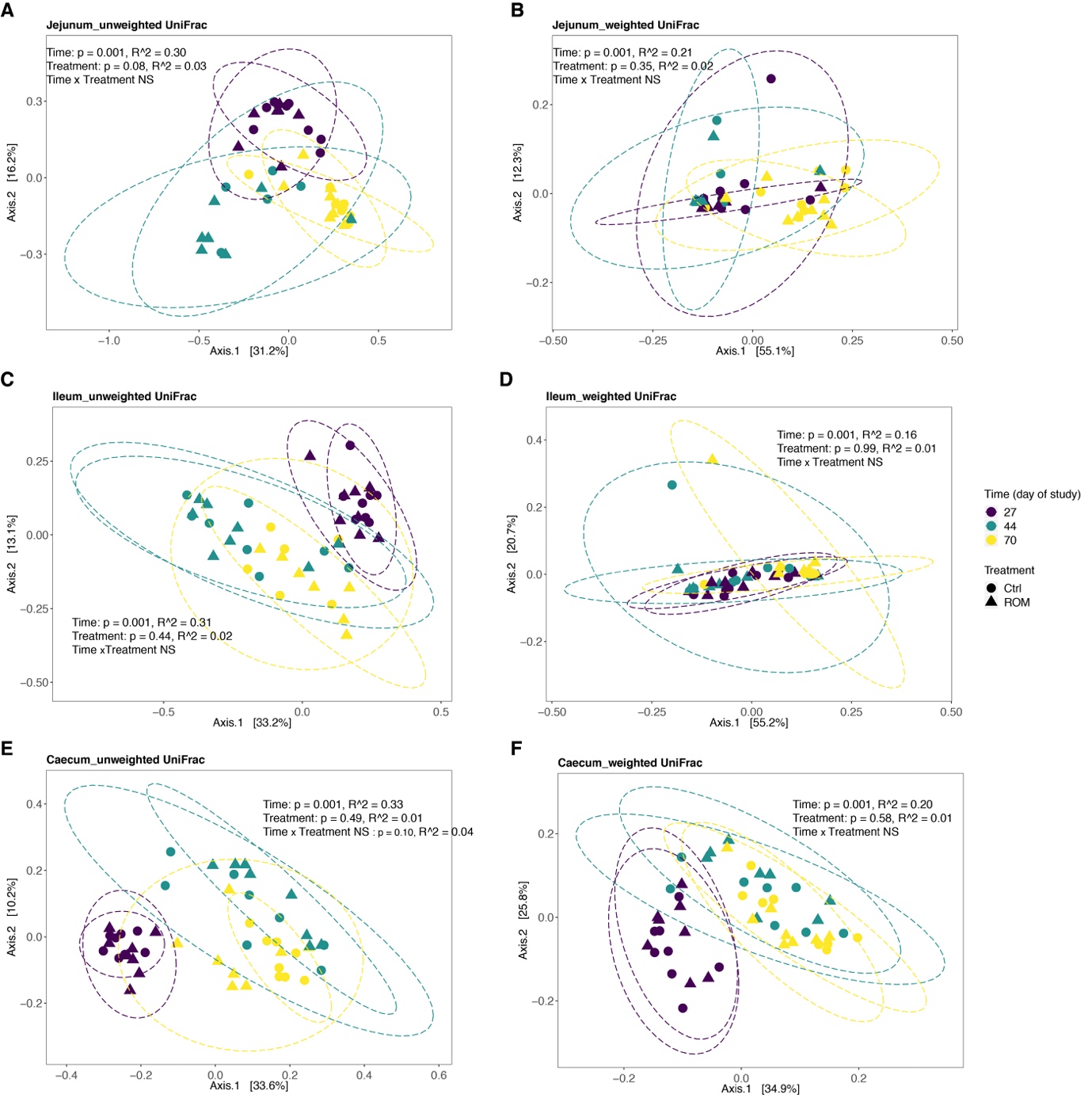


**Fig. S6 Principal coordinate analysis (PCoA) plots for the overall microbial composition of intestinal luminal digesta at different locations, based on unweighted- and weighted UniFrac metrics.** Significance of the effect of time and treatment on jejunal- (**A, B**), ileal- (**C, D**) and caecal (**E, F**) luminal microbiota at ASV level was assessed by PERMANOVA. Colours represent different time points, and shapes represent different treatment groups (circles, ROM; triangles, Ctrl). The percentages at the axes indicate the variation explained.


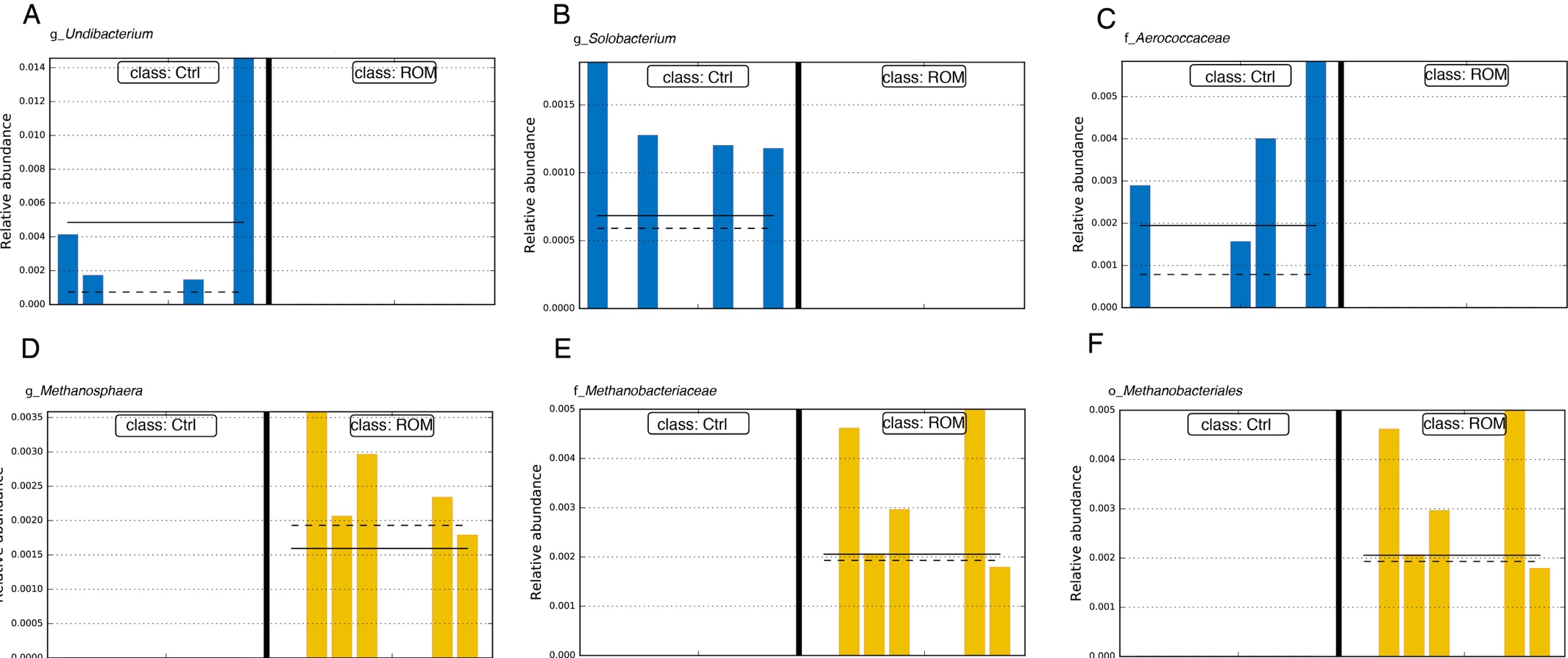


**Fig. S7 Bar plots representing relative abundance in individual pig jejunal (A-C) and caecal (D-F) luminal contents of specific taxa identified as differential features by LEfSe for pigs between control (class: Ctrl) and treatment (class: ROM) on day 70.** The solid black horizontal line indicates the mean relative abundance within each class.

**
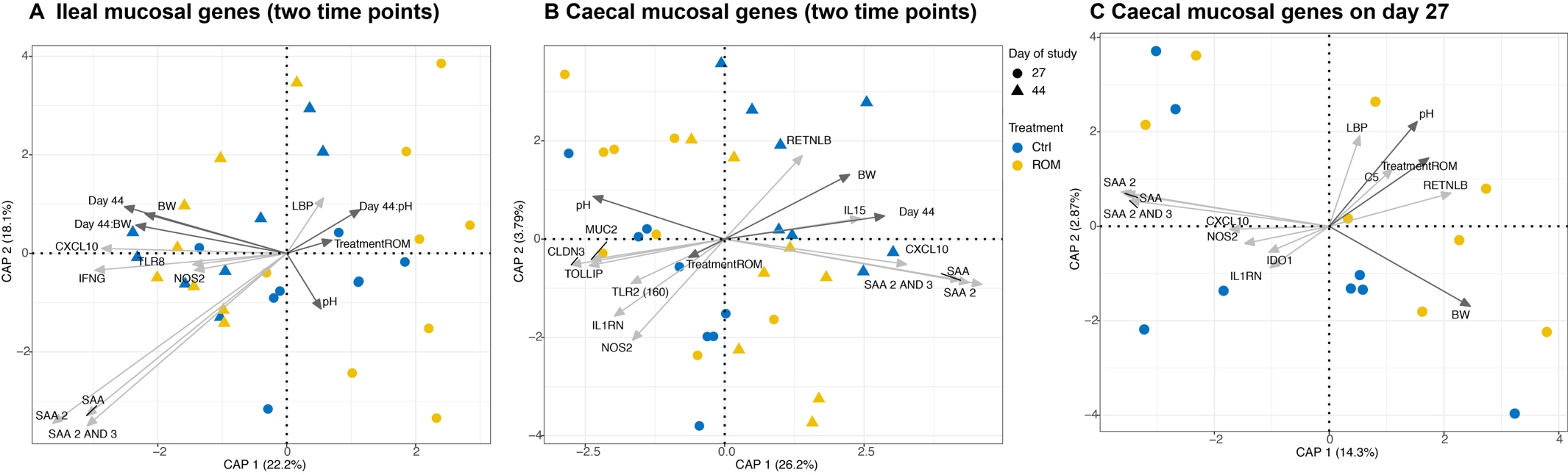
Fig. S8 Redundancy analysis (RDA) tri-plots for the association between the variation of gene expression and environmental variables of time, treatment, pH and BW.** (**A, B**) illustrates the association between ileal and caecal mucosa expressed genes with environmental variables of time, treatment, pH value and body weight (BW), respectively. (**C**) shows the association between the caecal mucosal gene expression and treatment, pH values and BW. Blue and yellow colours represent control (Ctrl) and treated (ROM) groups and shapes show the different time points. Dark grey arrows indicate environmental variables and light grey arrows show best fitting genes in the model. The percentages at the axes indicate the variation explained by the first two canonical axes.


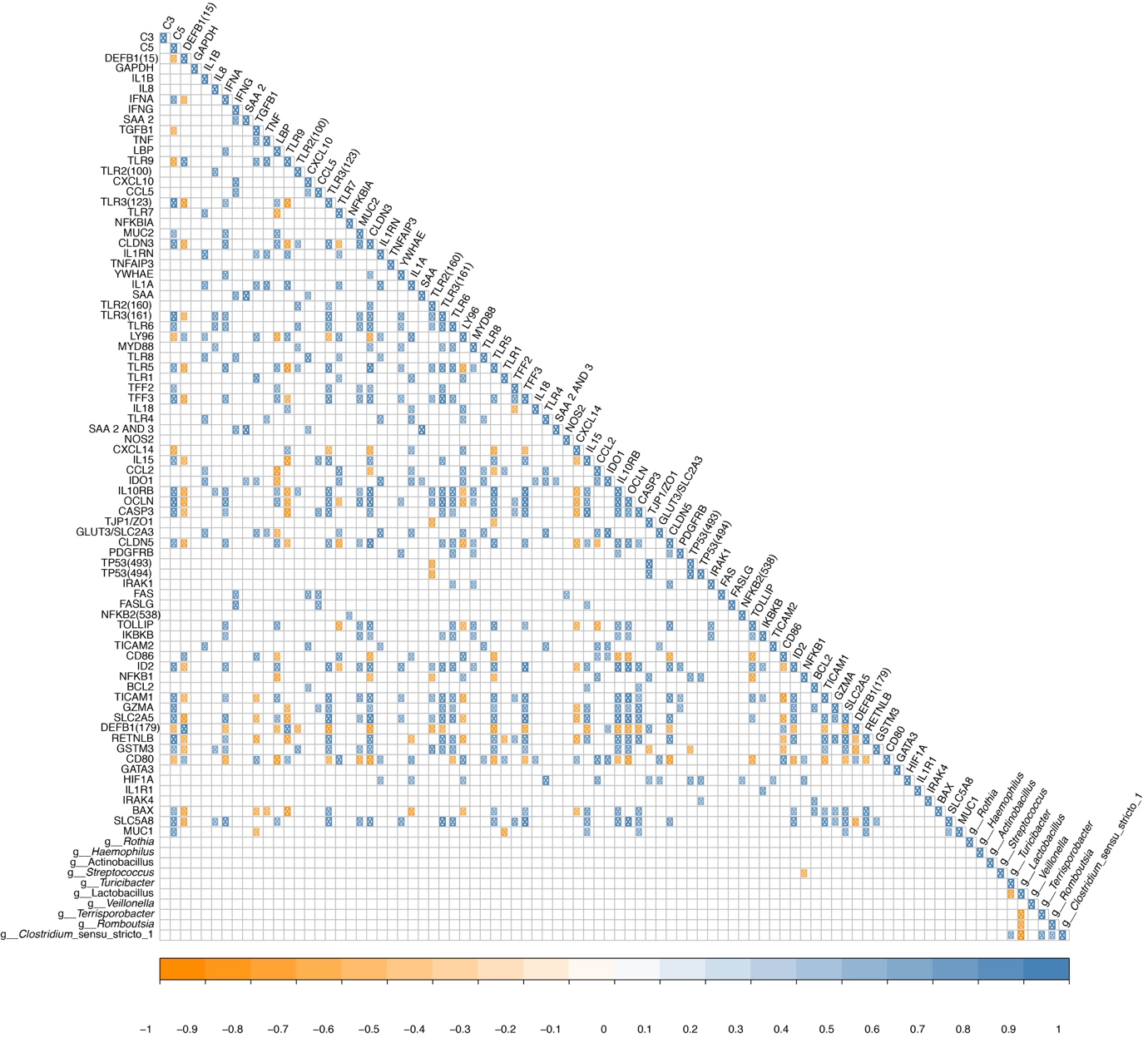


**Fig. S9** **Correlation analysis between the relative abundance of predominant ileal luminal genera and mucosal gene expression on day 27.** The orange and blue coloured circles correspond to a positive and negative statistical correlation with an adjusted p-value < 0.05, respectively. Insignificant correlations were shown as blank.

**
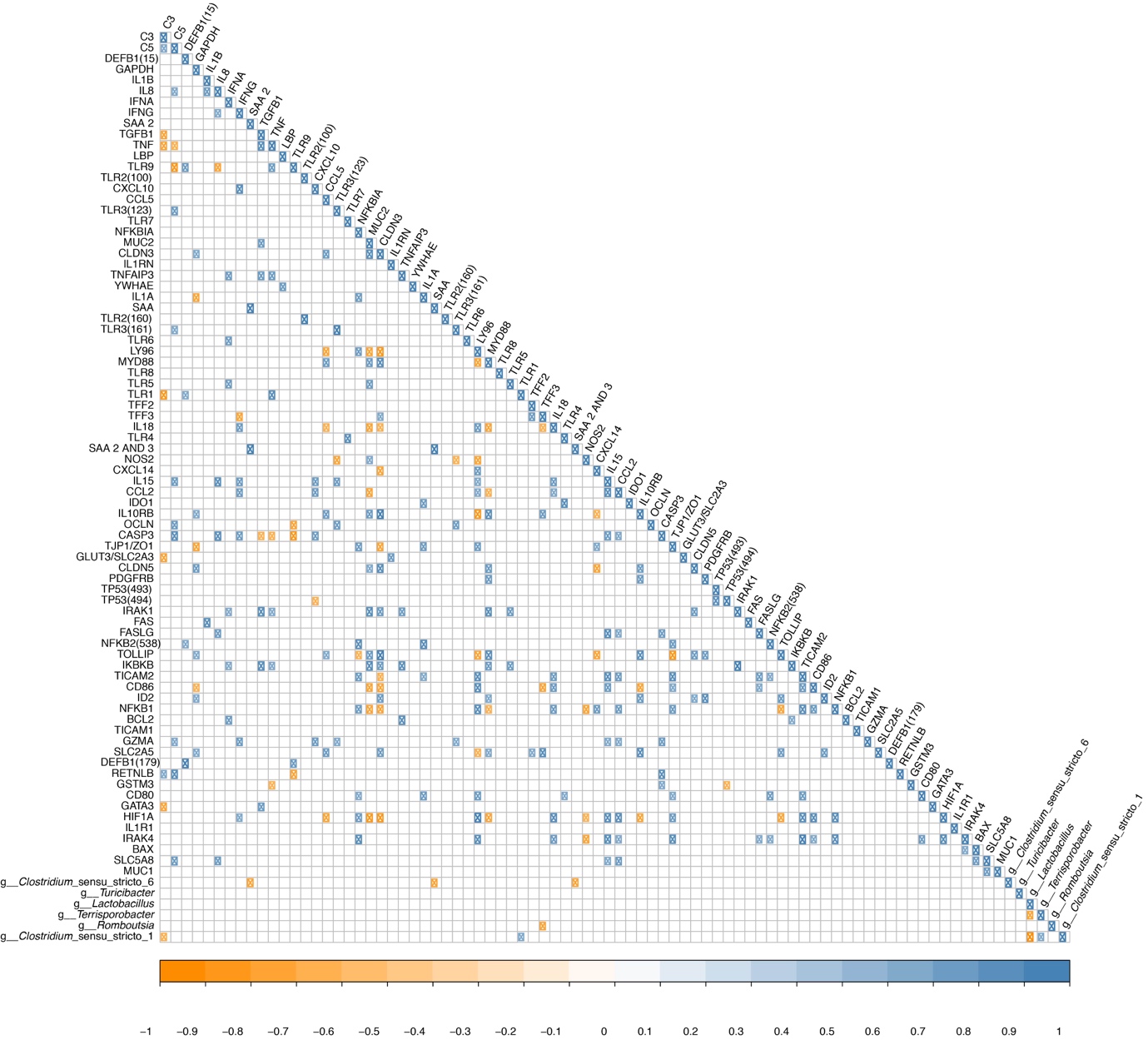
Fig. S10 Correlation analysis between the relative abundance of predominant ileal luminal genera and mucosal gene expression on day 44.** The orange and blue coloured circles correspond to a positive and negative statistical correlation with an adjusted p-value < 0.05, respectively.


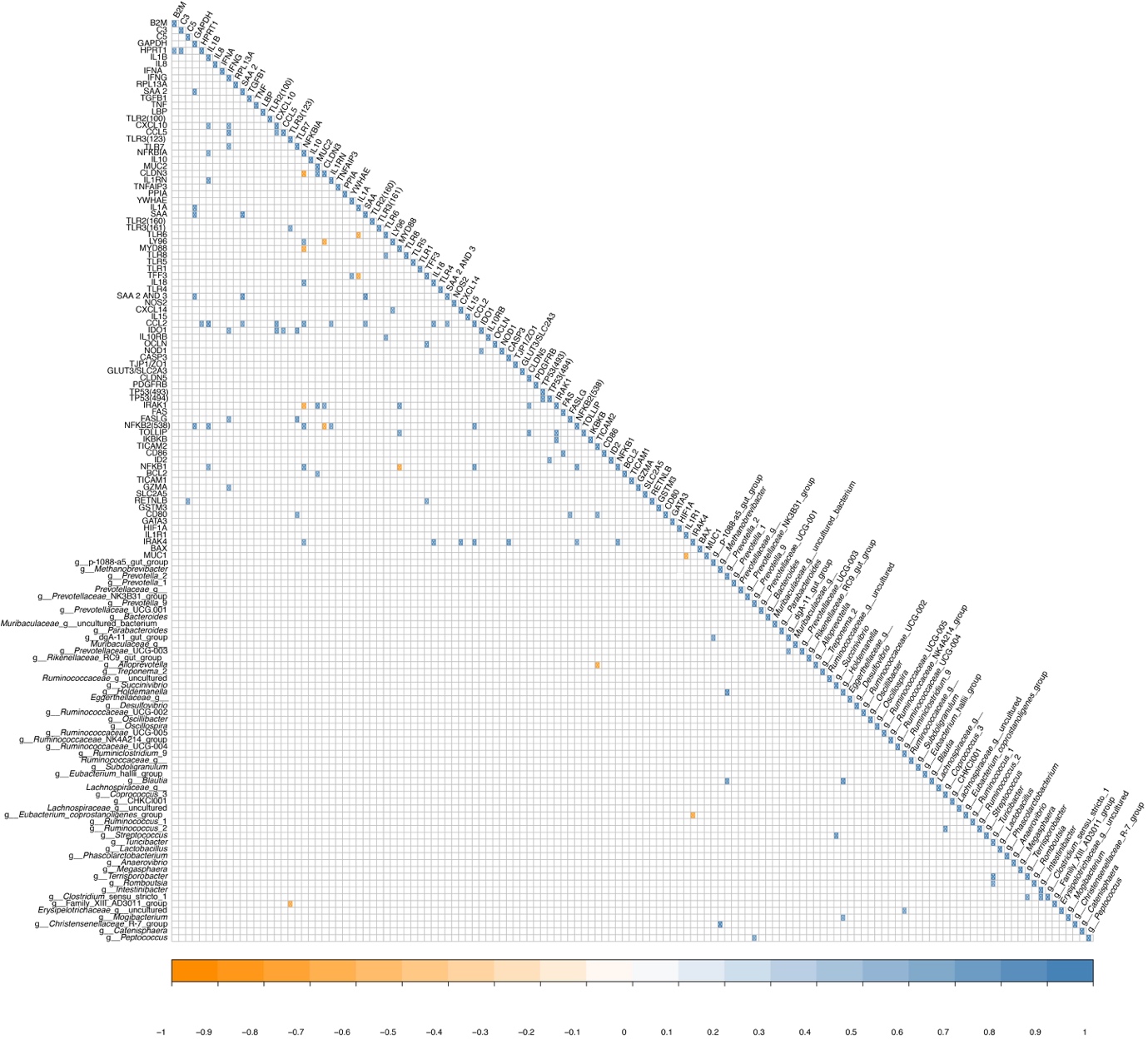


**Fig. S11** **Correlation analysis between the relative abundance of predominant caecal luminal genera and mucosal gene expression on day 27.** The orange and blue coloured circles correspond to a positive and negative statistical correlation with an adjusted p-value < 0.05, respectively. Insignificant correlations were shown as blank.


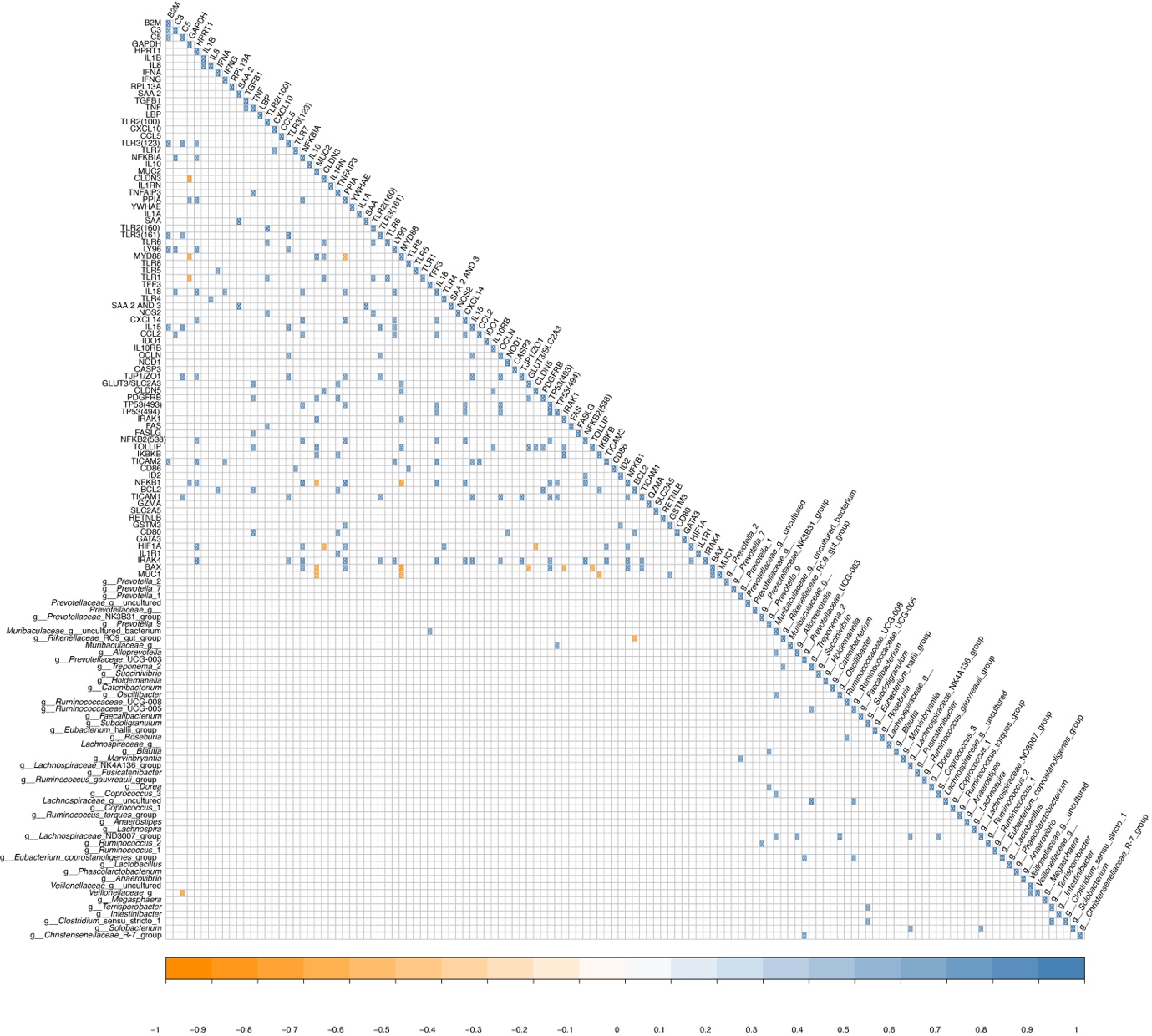


**Fig. S12** **Correlation analysis between the relative abundance of predominant caecal luminal genera and mucosal gene expressions on day 44.** The orange and blue coloured circles correspond to a positive and negative statistical correlation with an adjusted p-value < 0.05, respectively. Insignificant correlations were shown as blank
